# Combinatorial structure of iconic strategies in the manual-visual modality: evidence from silent gestures by hearing non-signers

**DOI:** 10.3389/fpsyg.2026.1917740

**Published:** 2026-10-16

**Authors:** Songlei Zhang, Hua Chen

**Affiliations:** 1 School of Foreign Studies, Nanjing University, Nanjing, China; 2 Department of Applied Foreign Language Studies, Nanjing University, Nanjing, China

**Keywords:** combinatorial structure, conceptual category, concreteness, iconicity, silent gesture

## Abstract

Iconicity, understood as perceived resemblance between aspects of form and meaning, provides a central basis for representing conceptual information in the manual-visual modality. Previous research on silent gesture has shown systematic associations between semantic categories and particular iconic strategies, with several strategies sometimes used together to represent a concept. How these strategies are organized when they are used together, however, remains less well understood. Using an individually administered task adapted from an established silent gesture paradigm, we elicited silent gestures from 38 Mandarin-speaking hearing non-signers to represent 246 concepts spanning concrete and abstract conceptual categories. We examined how concreteness and conceptual category were related to the occurrence of combinations and how meaningful manual units were organized sequentially, simultaneously, or in mixed structures. Our results show that most manual productions contained a single meaningful unit. Concrete concepts were more likely than abstract concepts to elicit combinations overall and simultaneous-only combinations, while conceptual category was associated with all three organizational formats. Sequential combinations were the most frequent, with drawing and embodying tending to precede acting, while pointing and schema mapping tended to precede symbolic mapping. Simultaneous combinations were characterized by recurrent pairings across the hands, especially acting with embodying and representing with locating, and broader iconicity-type pairings varied across conceptual categories. Mixed combinations were rare but clustered in concepts that invited both temporal staging and bimanual integration. Our findings extend patterned iconicity beyond the selection of individual form-meaning mappings to the relations among multiple iconic depictions. They show that the manual-visual modality provides temporal and spatial resources through which conceptual relations can be externalized and systematically organized even in the absence of an established sign language system.

## 1 Introduction

In the manual-visual modality, conceptual content can be expressed through motivated correspondences between meaning and bodily form, as the configuration and movement of the hands allow selected aspects of perceptual and motoric experience to be represented visually (Hassemer and Winter, 2018; Perniss et al., 2010, 2015). When such representations accompany speech, they contribute to multimodal utterances as co-speech gestures, whose timing and semantic content are closely coordinated with the spoken language (Kendon, 2004; Perniss et al., 2015). Their role in communication, however, extends to situations in which speech is unavailable and kinetic resources serve as the primary means of expression (Goldin-Meadow et al., 1996). Gesturers may trace an outline in the air, simulate a typical action, assign an entity to one hand, or use both hands to show how two components of a scene are related. Such productions, referred to as silent gestures, have been examined through the silent gesture paradigm, in which hearing individuals with no knowledge of a sign language are asked to convey meanings through gesture without speaking (Bradley and Wilbur, 2023; Flaherty and Schouwstra, 2025; Goldin-Meadow et al., 2008; Ortega and Özyürek, 2020a; Özçalışkan et al., 2016; Pouw et al., 2021). Research using this paradigm has revealed recurrent preferences in the ordering of constituents (Christensen et al., 2016; Goldin-Meadow et al., 2008; Hall et al., 2013; Schouwstra and de Swart, 2014) and the representation of event information (Bradley and Wilbur, 2023; Brentari et al., 2015; Slonimska et al., 2022), providing evidence of systematic organization in gestural expressions produced without accompanying speech (Goldin-Meadow et al., 2008; Hall et al., 2013; Özçalışkan et al., 2016). Related research has documented recurrent mappings between meaning and manual form that draw on the perceptual and motoric properties of the referent (Padden et al., 2015; van Nispen et al., 2017), with preferences for particular iconic form-meaning correspondences varying systematically across conceptual categories (Hwang et al., 2017; Mamus et al., 2025; Ortega and Özyürek, 2020a, 2020b).

A central basis for these form-meaning correspondences is iconicity, understood as perceived resemblance between aspects of a form and aspects of its meaning (Dingemanse et al., 2015; Emmorey, 2014; Hassemer and Winter, 2018; Ortega and Özyürek, 2020a; Perniss et al., 2010). Such resemblance can be established through a hand configuration that depicts a referent’s visible properties or through a movement that simulates an action associated with it, allowing perceptual and motoric information to enter the relation between gesture and meaning (Hwang et al., 2017; Ortega and Özyürek, 2020a, 2020b; Padden et al., 2015; van Nispen et al., 2017). These resemblances, however, are selective, as particular features of the conceptual representation are brought into correspondence with selected properties of the hands and their movements (Hassemer and Winter, 2018; Taub, 2001). A *ball*, for example, can be depicted by tracing its outline or by simulating a throwing action, with each form foregrounding a different aspect of the concept (Ortega and Özyürek, 2020a). Recurring ways of establishing these selective correspondences, described as iconic strategies or iconic representation modes, distinguish how conceptual features are mapped onto the hands and body.

A related question concerns how iconic representations are organized when several meaningful gesture units contribute to the expression of a concept. Studies of object pantomime have documented responses in which different depiction techniques are combined (van Nispen et al., 2017), while comparisons of action and object concepts have shown that objects more often elicit multiple gestures, including combinations of iconic depictions and pointing (Ortega and Özyürek, 2020b). More recent work has recorded both sequential and simultaneous depictions in responses to object and animal concepts (Mamus et al., 2025). These findings establish that several representations can contribute to a response and provide a basis for examining how the meaningful gesture units that express them are related. The occurrence of several units, however, leaves open whether particular units tend to precede others or to be paired with them across the hands.

Importantly, relations among gesture units can themselves participate in the representation of meaning. When gestures unfold sequentially, their order may reflect relations among the represented elements, as in descriptions of events in which the existence of an object depends on the action being represented (Christensen et al., 2016; Schouwstra and de Swart, 2014). Temporal order, however, is only one means of relating these contributions. In a simultaneous expression, one hand may depict an entity while the other represents an action involving it, so that their relative configuration and movement become part of the depiction (Bradley and Wilbur, 2023; Slonimska et al., 2022). Such bimanual relations illustrate the possibility of diagrammatic iconicity, in which relations among forms correspond to relations among their referents (Hodge and Ferrara, 2022; Perniss, 2007; Taub, 2001). When a simultaneous pairing occurs within a sequence, the resulting response brings these two forms of organization together, raising the further question of how they are coordinated within a single expression. Conceptual properties may help explain when several depictions are combined and how their contributions are related. Research on co-speech gesture, for instance, has linked the depiction of objects to their perceptual and action properties (Masson-Carro et al., 2016, 2017), while studies of abstract concepts have identified differences in sensorimotor content (Harpaintner et al., 2018) and in the contribution of social, affective, and linguistic experience (Villani et al., 2019). These findings motivate attention to conceptual categories alongside the broader distinction between concrete and abstract concepts. Here, we ask how hearing non-signers combine iconic strategies in silent gesture. We characterize each meaningful gesture unit in terms of five broad iconicity types, namely, action-based, form-based, relation-based, convention-based, and schema-based. We then ask how these resources are organized when more than one is recruited within a single response. We focus on three types of combinatorial structures: sequential, simultaneous, and mixed combinations. Specifically, we ask whether iconic strategy combinations vary with concreteness and conceptual category, whether different conceptual categories favor sequential, simultaneous, or mixed combinations, and whether particular strategies show recurrent patterns in sequential position, ordered transition, hand role, or bimanual pairing.

## 2 Literature review

### 2.1 Iconicity and iconic representational strategies

Iconicity, defined as the perceived resemblance between form and meaning, has been increasingly recognized as a central property of language and communication, challenging the traditional view that language is organized and defined primarily by arbitrary form-meaning mappings (Dingemanse et al., 2015; Emmorey, 2014; Perniss et al., 2010). Research on iconicity has distinguished the degree of perceived resemblance from the different ways in which that resemblance is established (Dingemanse et al., 2020; Ortega et al., 2025). In gesture, this latter question concerns which features of a conceptual representation correspond to which properties of the hands and body (Emmorey, 2014; Hassemer and Winter, 2018; Taub, 2001). The manual-visual modality offers a set of affordances. Hands can trace outlines, enact actions, represent entities, locate objects in space, and configure relations between referents. The body can stand for another body, a hand can stand for a handled object, and gesture space can stand for a conceptual or physical space (Fay et al., 2014; Frederiksen, 2017; Hodge and Ferrara, 2022; Nyst et al., 2022). In this sense, the manual-visual modality provides an especially rich domain for examining how form and meaning can be motivated through perceptual, motoric, and spatial resemblance. Differences in what the hands and body represent underlie the distinctions drawn between iconic strategies (Hwang et al., 2017; Ortega and Özyürek, 2020a).

Iconic mappings are selective, conventionalizable, and dependent on different representational resources: producers must choose which aspect of a concept to represent and how that aspect should be mapped onto bodily form (Dingemanse et al., 2015; Emmorey, 2014; Taub, 1997, 2001; van Nispen et al., 2017; Zlatev et al., 2020). Classifications of iconic strategies describe recurring forms of depiction and provide a basis for examining their distribution across conceptual categories. Müller (2014) conceptualized representational gestures as a set of depiction techniques in which the hands imitate actions, portray objects, mold shapes, or draw outlines. Padden et al. (2013, 2015) also developed the notion of patterned iconicity to describe the systematic association of particular iconic strategies with groups of referents that share semantic properties.

Existing studies have found that different semantic categories systematically recruit different representational resources. Research on silent gesture showed strong preferences for acting when expressing actions and manipulable objects, while drawing is particularly associated with non-manipulable objects (Ortega and Özyürek, 2020a, 2020b). Comparable differences have also been reported in co-speech gesture, where highly manipulable objects elicit more imitation of actions and objects that afford less manipulation more often elicit depictions of shape, including molding (Masson-Carro et al., 2016, 2017). In contrast, objects that do not allow for clear manual manipulation are more often represented through drawing and tracing (Hou, 2018; Keränen, 2021; Kimmelman et al., 2018; Masson-Carro et al., 2016; Ortega et al., 2025; Ortega and Özyürek, 2020a), or through molding (Masson-Carro et al., 2017). Other conceptual categories show different but equally systematic tendencies. Animals reliably recruit personification (Hwang et al., 2017; Ortega et al., 2025; Ortega and Özyürek, 2020a), in which the signer’s or gesturer’s body is mapped onto the body of the animal referent. They may also be represented through object or entity strategies when their bodies are difficult to map onto the human body (e.g., small insects and some legged creatures) (Hou, 2018; Schiefner, 2025). Preferences of this kind have been observed in both blind and sighted gesturers, with visual experience nevertheless associated with differences in the use of drawing and personification (Mamus et al., 2025).

Importantly, research on iconic strategies is not limited to highly concrete concepts. For meanings with no stable visual form, iconic representations can draw on bodily experience and on associations with more readily depicted referents (Borghi et al., 2014; Meir, 2010). In Finnish Sign Language, lexical signs referring to sensory and emotional meanings (i.e., touch, perception, and emotion) frequently use hand-action and entity strategies, extending these mappings to information associated with bodily sensation (Keränen, 2021). In their study of iconicity and concreteness in British Sign Language and German Sign Language, Ortega et al. (2025) discuss how abstract meanings can be represented through their association with concrete referents. The British Sign Language sign for *court*, for example, traces the shape of a barrister’s wig, establishing an iconic depiction of an object that is metonymically associated with the institution. More generally, when a concept cannot be directly depicted through visual similarity, representation may shift toward less direct routes such as semantic association, metonymy, metaphor, body language, schematic structure, or convention (Meir, 2010; Nakamura and Zeng-Treitler, 2012; Jo, 2026). Cultural conventions also contribute to the interpretation of familiar gestures (Gawne and Cooperrider, 2024), whose conventional meanings may coexist with iconic relations to their referents (Hodge and Ferrara, 2022).

Across these accounts, distinctions between representational strategies depend on both the properties selected for depiction and the breadth of the categories used to describe them. For example, Hwang et al. (2017) place both handling and instrument forms within manipulation, as both depict a human action involving an object. Ortega and Özyürek (2020a), by comparison, distinguish acting from representing according to whether the hands depict bodily action or take on the form of the referent. This variation in terminology also motivates the present study. We distinguish between fine-grained strategies and broader iconicity types. At the strategy level, coding identifies the specific correspondence between form and meaning expressed by a meaningful gesture unit, while at the type level it identifies the broader relation shared by strategies drawing on similar representational resources. This distinction allows us to align the present analysis with previous work while avoiding an overly fragmented coding scheme. We group iconic representation strategies into five broader types: Action-based, Form-based, Relation-based, Convention-based, and Schema-based iconicity. The extensions involving symbolic mapping and schema mapping were developed after inspecting the productions, with their interpretation informed by research on conventional gestures and schematic representation. Operational definitions and coding criteria for all categories are provided in Section 3.4.

### 2.2 From iconic strategies to combinatorial structure

Several iconic depictions can contribute to the expression of a concept, with different gestures representing complementary aspects of its meaning. In their study of object pantomime, van Nispen et al. (2017) found that some objects were consistently depicted through combinations of representation techniques. Pantomimes of *cactus*, for example, included depictions of its shape together with gestures of touching a thorn and reacting to pain. In such responses, the object’s visible form is represented alongside an interaction associated with it, allowing the contribution of each depiction to be distinguished within the larger expression. Comparisons across conceptual categories have further clarified when such combinations are produced and what information they convey. Ortega and Özyürek (2020b) found that object concepts are more likely than action concepts to elicit combinations of iconic strategies. Using gestures from the production task, they then asked a separate group of viewers to identify the intended meanings and found greater accuracy for object depictions containing two gestures than for those containing only one. These findings show that combinations are sensitive to conceptual distinctions and that their communicative contribution can be investigated independently of their occurrence.

The arrangement of these iconic depictions, however, warrants consideration alongside the strategies they express. In their comparison of gestures produced by blind and sighted participants, Mamus et al. (2025) recorded both sequential and simultaneous depictions of objects and animals, including combinations of manual and nonmanual components. A closer analysis can distinguish the occurrence of particular strategies from their relative positions and pairings within these expressions. When relations among gesture units correspond to relations among their referents, the expression can exhibit diagrammatic iconicity, through which the arrangement of forms contributes to the depiction of a conceptual relation (Hodge and Ferrara, 2022; Perniss, 2007; Taub, 2001). For this reason, iconic strategy combinations invite analysis not only as co-occurring resources, but as organized structures whose temporal and spatial relations may contribute to the construal of the target concept.

A broader vocabulary for describing this organization comes from sign language research. Established sign languages make extensive use of both sequential and simultaneous organization, distributing meaningful elements across time or layering them across articulators (Aronoff et al., 2005; Brentari, 2002; Loos et al., 2022; Meier, 2012; Morgan, 2014; Vermeerbergen et al., 2007). Sequential organization has been discussed in relation to compounds, complex predicates, resultatives, and the segmentation of event components such as manner and path (Brentari et al., 2024; Fischer and Janis, 1990; Senghas et al., 2004; Supalla, 1982, 1990). Simultaneous organization draws on the availability of multiple articulators, allowing different components of meaning to be expressed within the same temporal window (Brentari, 2002; Cormier et al., 2015; Dudis, 2004; Slonimska et al., 2022). Handshape, movement, location, body posture, facial expression, gaze, and the relative configuration of the two hands may all participate in the encoding of event or relational information (Slonimska et al., 2020). Established sign languages exploit these resources within conventional grammatical systems (Brentari et al., 2020, 2024; Cormier et al., 2013; van der Kooij et al., 2023; Lepic et al., 2016; Loos et al., 2022). Silent gesture lacks that linguistic conventionalization, but the bodily and spatial medium remains available to non-signers. The two hands, in particular, can be used to place different pieces of information in the same moment of expression (Bradley and Wilbur, 2023; Goldin-Meadow et al., 1996; Slonimska et al., 2020; Slonimska et al., 2022). Slonimska et al. (2022) found that both Italian silent gesturers and LIS signers increased simultaneity as information density increased, while silent gesturers used fewer simultaneous resources than signers. Börstell and Lepic (2025) further show that hearing non-signers favor two-handed gestures for inherently plural meanings, pointing to a broader association between paired articulators and plural or relational construals. These findings show that hearing non-signers use simultaneous expression, although its frequency and form differ from those observed in established sign languages.

### 2.3 The present study

In the present study we examine how hearing non-signers combine iconic strategies in silent gesture production. We use silent gesture as a window onto the organization of visible meaning because participants must communicate without speech and without access to an established sign language system (Flaherty and Schouwstra, 2025; Ortega and Özyürek, 2020a; Özçalışkan et al., 2016; Pouw et al., 2021). We examined manually articulated units in a production dataset elicited from 38 Mandarin-speaking hearing non-signers for a broad set of concrete and abstract concepts. The inclusion of abstract concepts is central to the present design. Research on silent gesture combinations has primarily examined actions and objects (Ortega and Özyürek, 2020b; van Nispen et al., 2017). Studies of lexical signs further show that iconic strategies can extend to sensory, emotional, and other abstract meanings (Keränen, 2021; Ortega et al., 2025). Abstract concepts may pose different representational demands because they often lack a stable visible referent, and may depend more heavily on action schemas, spatial relations, conventional associations, or schematic mappings (Borghi et al., 2014; Conca et al., 2021). Thus, examining both concrete and abstract concepts allows us to ask whether iconic strategy combinations are primarily tied to imageable meanings, or whether similar organizational formats also appear when the target meaning is less directly depictable.

We pursue three related research questions. First, how are concreteness and conceptual category related to the occurrence of gesture combinations, both overall and for sequential, simultaneous, and mixed combinations? Second, which iconic strategies recur together across the hands in simultaneous combinations, and how do pairings of the broader iconicity types vary across conceptual categories? Third, how are iconic strategies distributed across positions in sequential combinations, and do recurrent pairs show preferred temporal orders? Mixed combinations further allow us to consider how sequential and simultaneous components are coordinated within one expression. Through these questions, we seek an empirical account of how the iconic contribution of each meaningful gesture unit relates to the other units with which it is expressed. This extends the study of patterned iconicity to the organization of combinations, linking conceptual differences with recurring relations among their components. To capture organizational patterns within combinations of iconic strategies, we analyze the internal structure of combinations by examining sequential position, ordered transitions, hand role, and bimanual pairing.

## 3 Method

### 3.1 Participants

We initially recruited 40 hearing adult native speakers of Mandarin through online advertisements at Nanjing University. All participants were undergraduate, master’s, or doctoral students currently enrolled at the university. None reported prior knowledge of Chinese Sign Language or any other sign language. They had also received no formal training in gesture, sign language, or other multimodal communication systems. All participants had spent most of their lives in mainland China, and none had a history of extended residence abroad. English was the second language of all participants, and none reported advanced proficiency in any additional spoken language. The recruitment was restricted to right-handed participants to reduce variation associated with handedness. All reported normal or corrected-to-normal vision and no motor condition that would interfere with movements of the hands or upper body. Two participants were excluded because they produced analyzable silent gestures for fewer than 30% of the 246 target concepts (21 and 23%, respectively). The final sample therefore included 38 participants (30 female; *M* = 23.87 years, *SD* = 2.91, range = 18–29). Participants provided written informed consent, agreed to be video-recorded, and received 60 RMB for their participation.

### 3.2 Stimuli

The stimulus set consisted of 246 concepts presented as Chinese lexical items, including 162 concrete concepts and 84 abstract concepts. We compiled the initial pool of 500 Chinese lexical items from SUBTLEX-CH, a database of Chinese word and character frequencies based on film and television subtitles (Cai and Brysbaert, 2010), giving preference to relatively high-frequency words. Function words, highly polysemous items, and strongly context-dependent items^1^ were excluded, as were items that required specialist knowledge or additional linguistic context to identify the intended concept. The selection also aimed to maintain broad coverage across the conceptual categories while limiting close semantic overlap within individual categories.

Next, an independent group of 26 undergraduate students at Nanjing University (14 female, aged 17 to 22 years) was recruited through online campus advertisements to rate the concreteness of the candidate items on a 7-point scale from 1 (highly abstract) to 7 (highly concrete). All raters were native speakers of Mandarin who had spent most of their lives in mainland China and had no history of extended residence abroad. English was their second language, and none reported advanced proficiency in any additional spoken language. None took part in the silent gesture elicitation task. The ratings showed good internal consistency (Cronbach’s α = 0.84). Items with a mean rating of 3 or lower (*N* = 105) were classified as abstract (*M*_rating_ = 2.24, *SD* = 0.28), whereas items with a mean rating of 5 or higher (*N* = 192) were classified as concrete (*M*_rating_ = 5.88, *SD* = 0.59). The retained candidate items (*N* = 297) were then classified into six concrete and six abstract conceptual categories. We adapted five concrete categories from Ortega and Özyürek (2020a) and added social role entities to include socially defined human roles. The six abstract categories included emotions, social concepts, cognition, human actions, attributes, and magnitude-related concepts, and were selected with reference to previous classifications in psycholinguistics and cognitive neuroscience (Borghi et al., 2017; Conca et al., 2021; Harpaintner et al., 2018). The first author and two postgraduate students in linguistics, all native speakers of Mandarin, independently assigned the candidate items to the 12 categories after reviewing definitions and examples for each category. Inter-coder agreement was near perfect (Fleiss’ κ = 0.96, *p* < 0.001). Items with persistently unclear category membership or close semantic overlap were excluded, and larger category pools were reduced to avoid over-representation of a small number of concept types.

The final stimulus set consisted of 246 concepts across 12 conceptual categories: actions with objects (*N* = 30), actions without objects (*N* = 21), manipulable objects (*N* = 44), non-manipulable objects (*N* = 36), animate entities (*N* = 21), social role entities (*N* = 10), emotions (*N* = 15), social concepts (*N* = 15), cognition (*N* = 13), human actions (*N* = 17), attributes (*N* = 12), and magnitude-related concepts (*N* = 12) (Table 1). The complete stimulus list is provided in Supplementary Material S1. The number of items varied across categories, reflecting differences in the number of concepts that met the selection criteria.

**Table 1:** Conceptual categories, numbers of items, and examples.

Concreteness	Conceptual category	*N*	Example
Concrete	Actions with objects	30	eating
Concrete	Actions without objects	21	singing
Concrete	Manipulable objects	44	mobile phone
Concrete	Non-manipulable objects	36	plane
Concrete	Animate entities	21	snake
Concrete	Social role entities	10	teacher
Abstract	Emotions	15	sadness
Abstract	Social concepts	15	friendship
Abstract	Cognition	13	intuition
Abstract	Human actions	17	war
Abstract	Attributes	12	kindness
Abstract	Magnitude-related concepts	12	volume

### 3.3 Procedure

We followed the silent gesture elicitation paradigm used in previous research with hearing non-signers (Ortega and Özyürek, 2020a, 2020b). Participants were tested individually in a quiet room at Nanjing University. Each participant sat in front of a 13-inch laptop, and a high-definition camera positioned directly in front of the participant recorded the gesture productions at 60 frames per second. During the formal task, the researcher remained in the room to ensure that the procedure was followed but did not provide feedback on gesture production. Before the task, participants provided informed consent, completed a brief demographic questionnaire, and read the task instructions. They were told that they would see Chinese words one at a time and that their task was to communicate each word using silent gestures only. Participants were also told that their gestures would later be shown to other people, who would try to guess the intended meanings. We also noted that there were no correct or incorrect ways to express a concept. Participants then completed five practice trials to familiarize themselves with the procedure. Procedural feedback was provided during practice to ensure that the task requirements were understood. During the task, participants were not allowed to speak, point to objects in the room, or stand up before completing the experiment. If they were unable to produce a gesture for a target concept, they could say “pass,” after which the trial continued automatically.

The experiment was programmed using PsychoPy (Peirce et al., 2019). Each trial began with a fixation cross presented at the center of the screen for 500 ms. A target word was then displayed in black font on a white background for 4,000 ms, during which participants were required to produce silent gestures to convey the word’s meaning. We adopted the interval of four seconds used by Ortega and Özyürek (2020a) to encourage an immediate, spontaneous response. A new trial began automatically after the 4,000 ms interval. Stimuli were presented in a randomized order for each participant. Participants were allowed to take breaks during the session. The complete session, including instructions, practice trials, the formal task, and breaks, lasted approximately 30 min.

### 3.4 Coding

The coding proceeded in three steps using ELAN (Wittenburg et al., 2006). We first identified meaningful gesture units, then assigned each unit an iconic strategy and its corresponding iconicity type, and finally classified each response according to the organization of its meaningful units. The coding scheme drew on previous classifications of iconic representation in gesture and sign language and was refined to accommodate recurrent forms in the present data (Hwang et al., 2017; Ortega and Özyürek, 2020a, 2020b; van Nispen et al., 2017).

#### 3.4.1 Meaningful gesture units

The first step involved the identification of meaningful gesture units. We defined a meaningful gesture unit as the smallest manually articulated component that contributed identifiable semantic content to a response. For units produced in succession, boundaries were identified from changes in representational content and the completion of one semantic contribution before another began. Transitional movements, preparation movements, returns to a resting position, self-adjustments, hesitations, and repetitions that added no semantic content were not coded as separate units. Repeated or similar movements were coded separately only when they contributed distinguishable semantic information. Facial expression, gaze, head movement, and body posture accompanying a manual gesture were treated as part of the broader response and were not coded as independent meaningful units. A single response could therefore contain one unit, several units produced in sequence, or multiple units produced simultaneously when the two hands encoded distinguishable aspects of meaning. Movements that were abandoned before an identifiable semantic contribution was completed were treated as hesitation or preparation and were not counted as separate units. When one completed meaningful unit was followed by another unit contributing distinguishable semantic information, both units were retained in the coding. Because repair intention could not be determined independently from the production itself, completed movements were segmented according to their observable semantic contribution rather than classified separately as corrections. Segmentation rules and worked examples are provided in Sections 2.1 and 8 of the Supplementary Codebook.

#### 3.4.2 Iconic strategies and iconicity types

Previous classifications of iconic representation have distinguished recurring ways in which the hands and body establish correspondences with meaning, while also recognizing that more specific representational forms may share broader properties (Müller, 2014; Padden et al., 2015; Hwang et al., 2017; Ortega and Özyürek, 2020a). Hwang et al. (2017), for example, grouped more specific forms when they shared a common representational property, such as the involvement of the body as a human agent or the use of the hands to represent an entity. Following this general approach, we coded iconic representation at two related levels, distinguishing 11 iconic strategies and five broader iconicity types.

The two levels served different purposes in our analysis. At the more specific level, iconic strategy identified how a meaningful gesture unit established a particular form-meaning correspondence, while iconicity type captured the broader form-meaning relation shared by strategies that drew on a similar representational resource. The strategy level preserved distinctions between specific ways of representing meaning, allowing us to distinguish, for example, acting from enacting or drawing from representing. The type level allowed us to examine whether these more specific strategies participated in broader patterns when they relied on comparable form–meaning relations. This distinction was particularly important to the analysis of simultaneous combinations, where we first examined how broader types of iconic representation were distributed across the two hands and then examined which specific iconic strategies contributed to those patterns. In this way, the two levels allowed us to characterize both the general organization of iconic representation and the specific strategies through which that organization was realized. Table 2 summarizes the five iconicity types, the 11 iconic strategies, and the principal criteria used to assign each strategy.

**Table 2:** Iconicity types, iconic strategies, and coding criteria.

Iconicity type	Iconic strategy	Coding criterion
Action-based	Acting	The hand(s) simulate a specific manual action or object-directed manipulation.
Action-based	Enacting	The hands participate in the reenactment of a broader activity, bodily state, or emotional experience.
Form-based	Drawing	The hand dynamically traces an outline or visible contour of the referent in the air.
Form-based	Embodying	The hand(s) take on the form or structural configuration of the referent.
Form-based	Representing	The hand functions as a proxy or token for an entity, object, person, or participant in a represented scene.
Form-based	Personification	The gesturer’s body is mapped onto the body or body part of a non-human referent.
Relation-based	Pointing	The hand indicates a spatial position, direction, or imagined referential locus.
Relation-based	Locating	The hand establishes the position of an entity or provides a ground or surface relative to which another entity is depicted.
Relation-based	Path mapping	The hand traces a trajectory, route, or movement path.
Convention-based	Symbolic mapping	The gesture uses a culturally familiar form–meaning association with iconic motivation.
Schema-based	Schema mapping	The gesture depicts a generalized spatial or dynamic relation, such as containment, balance, scale, or recurrence, that corresponds to the target meaning.

The three iconicity types that included more than one strategy captured shared form–meaning relations across more specific realizations. **Action-based** iconicity included *acting* and *enacting*, which both relied on bodily and manual actions as the principal basis of the form-meaning correspondence. **Form-based iconicity** included *drawing*, *embodying*, *representing*, and *personification*, which drew on visible form or entity structure while differing in how that information was related to the hands or body. **Relation-based iconicity** included *pointing*, *locating*, and *path mapping*, which established meaning through spatial location, configuration, or trajectory. As coding proceeded, two recurrent sets of meaningful gesture units extended the scope of this initial inventory. One set drew on culturally recognizable manual forms whose contribution depended on a conventional association, while the other used recurrent spatial or dynamic structures to establish schematic relations with the target meaning. We therefore extended the coding scheme to include symbolic mapping and schema mapping as specific iconic strategies, grouped under convention-based and schema-based iconicity, respectively. These extensions were informed by established research on conventional gestures and emblems and on image-schematic organization in gesture (Kendon, 2004; Poggi, 2008; Cienki, 2013; Mittelberg, 2018).

The first of these extensions concerned culturally recognizable manual forms. Previous gesture research has commonly described such forms as emblems, quotable gestures, or codified symbolic gestures, emphasizing their relatively stable associations with shared meanings within a cultural community (Ekman and Friesen, 1969; Kendon, 2004; Poggi, 2008). Emblems constitute a broader class of culturally conventional gestures, and conventionalization does not exclude iconic or metaphorical motivation in their form-meaning relations (Gawne and Cooperrider, 2024; Hodge and Ferrara, 2022). In the present coding, symbolic mapping was restricted to culturally recognizable manual forms whose conventional meaning retained an identifiable iconic or metaphorical motivation. The positive meaning of a thumbs-up gesture, for example, has been related to the metaphorical association of upward orientation with positive evaluation (Gawne and Cooperrider, 2024). For gestures resembling actions, coding depended on whether the unit represented an enacted activity or contributed a conventional meaning. Clasped hands used as a conventional symbol of hope or gratitude were coded as symbolic mapping, whereas a reenactment of praying was coded as enacting. The term allowed these forms to be incorporated into the same mapping-based framework as the other iconic strategies while retaining the role of conventional association in their interpretation.

The second extension concerned gesture units whose spatial or dynamic organization itself carried a schematic relation to the target meaning. **Schema-based** iconicity involved making a less directly depictable meaning visible through a schematic structure. We introduced this category because some responses relied on recurrent schematic patterns such as containment, path, balance, force, scale, cycle, or center–periphery. Such patterns are widely discussed in cognitive linguistics as image schemas: embodied structures that arise from recurrent bodily and spatial experience and can organize meanings that are not themselves directly visible (Johnson, 1987; Lakoff and Johnson, 1980; Langacker, 2008; Talmy, 2000). In our study, *schema mapping* referred to gestures that represented the target concept through a schematic spatial or dynamic pattern.

To apply this category consistently, we distinguished schema mapping from neighboring strategies according to the relation represented by the gesture. Movements tracing the visible contour of an object in the air were coded as drawing, movements representing a concrete route or trajectory were coded as path mapping, and configurations placing an entity at a particular location were coded as locating. Culturally recognizable manual forms were coded as symbolic mapping when their conventional meaning retained an identifiable iconic or metaphorical motivation. Schema mapping was assigned when a schematic spatial or dynamic relation provided the principal correspondence with the target meaning. A circular movement, for example, was coded as schema mapping when it represented recurrence, as drawing when it outlined an object, and as path mapping when it depicted movement along a route. In addition, units that contributed identifiable semantic information but could not be assigned reliably to one of the 11 iconic strategies were coded as other. These units were retained in the response-level coding and excluded from analyses that required a valid iconic-strategy label. Representative examples of the 11 iconic strategies are shown in Figure 1. Additional examples and coding boundaries are provided in Section 4 of the Supplementary Codebook, with rules for units coded as other in Section 7.

**Figure 1:**
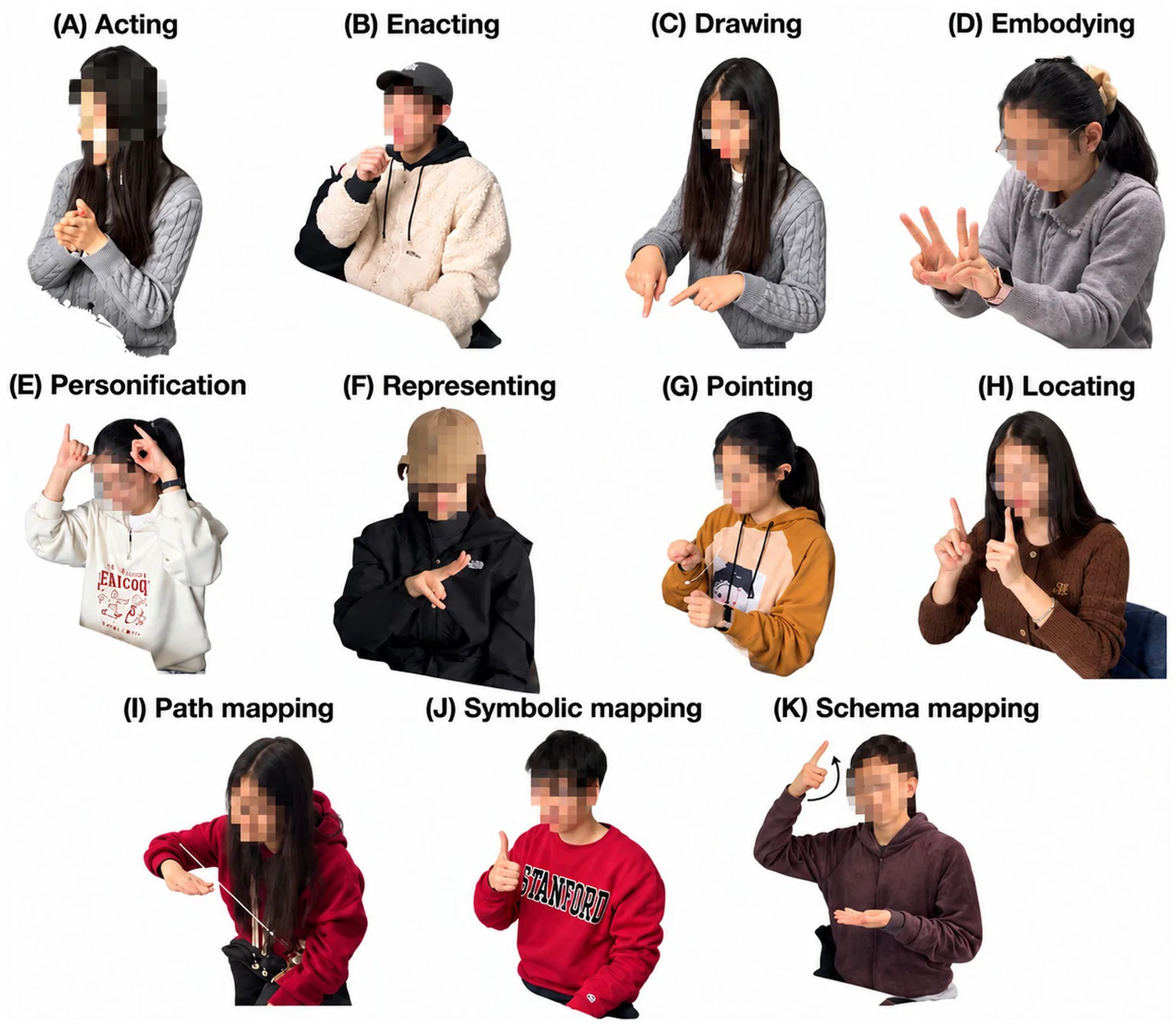
Examples of iconic strategies. Panels show examples of **(A)** acting for *soap*, **(B)** enacting for *singing*, **(C)** drawing for *frying pan*, **(D)** embodying for *rabbit*, **(E)** personification for *sheep*, **(F)** representing for *trimming*, **(G)** pointing for *center*, **(H)** locating for *suspicion*, **(I)** path mapping for *river*, **(J)** symbolic mapping for *intelligence*, and **(K)** schema mapping for *thinking*.

#### 3.4.3 Simultaneous, sequential, and mixed combinations

The third step involved classifying each silent gesture response according to the number and organization of its meaningful gesture units. A response was coded as non-combinatorial when it contained only one meaningful gesture unit. A response containing two or more meaningful gesture units was coded as combinatorial, including cases in which two semantically distinguishable units were assigned the same iconic strategy. Combinatorial responses were further classified as sequential-only, simultaneous-only, or mixed.

We coded a response as **simultaneous-only** when the two hands expressed distinguishable meaningful units within the same temporal interval and no larger sequential progression was present. Importantly, bimanual movement alone did not define a simultaneous combination. When both hands jointly formed one integrated depiction, the production was coded as a single meaningful unit. For example, both hands could jointly trace a heart shape to represent *love*, or contribute different parts of a single bodily representation of *monkey*. By contrast, a response was coded as simultaneous when the two hands encoded different aspects of the target meaning (Figure 2). For each simultaneous pairing, the two hands were coded as focal and anchoring according to their relation within that pairing. The focal hand carried the comparatively foregrounded or specifying unit, while the anchoring hand provided a comparatively stable or relational reference for the paired unit. Assignment was based on the relative movement of the two hands, their temporal stability, and the dependency between the two units. These labels described local functional positions within a pairing and did not refer to anatomical handedness or a stable specialization of either hand. Simultaneous pairings were recorded using the symbol “&,” with the focal-hand unit listed first and the anchoring-hand unit second. For hiking, one hand represented a walking figure with an inverted V-shaped configuration while the other provided the surface on which the figure moved (representing & locating, Figure 2A). To depict *suspicion*, for instance, one hand represented an entity while the other simultaneously pointed toward that entity, yielding two distinguishable contributions within the same response (pointing & representing, Figure 2B). Similarly, for stirring, one hand embodied a container while the other simulated a stirring action (acting & embodying, Figure 2C). The present analysis was restricted to manual simultaneity; accompanying facial, bodily, and gaze behaviors were not coded as additional simultaneous units.

**Figure 2:**
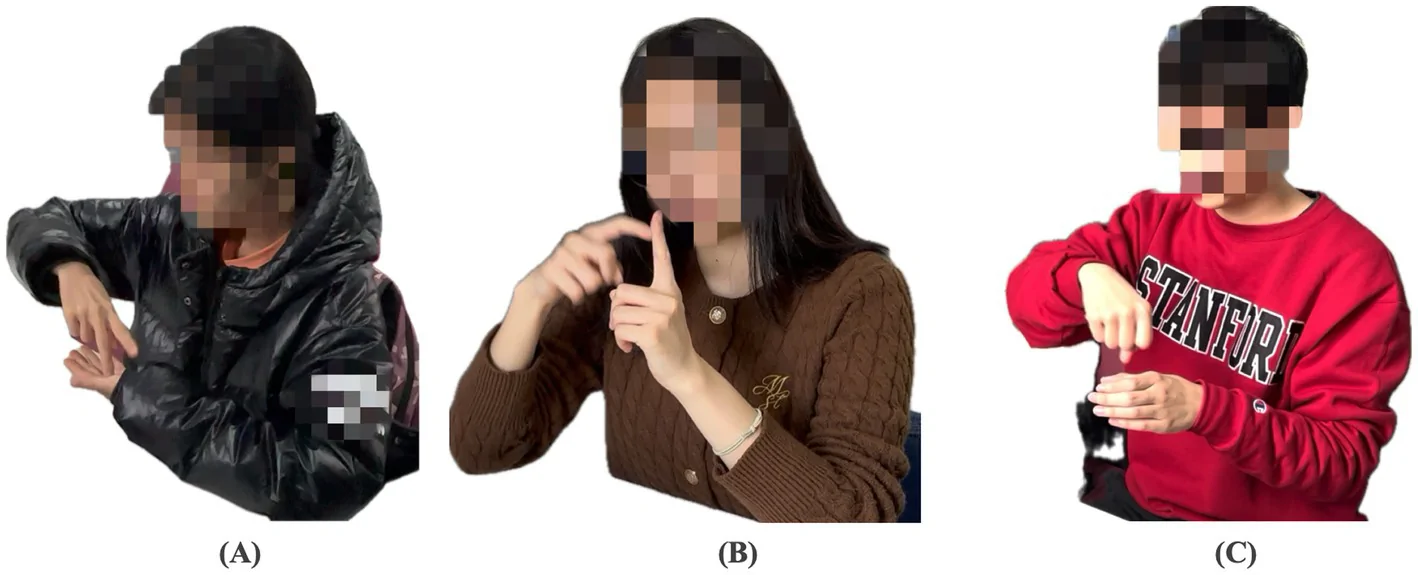
Examples of simultaneous-only combinations. **(A)** *hiking*, representing & locating. **(B)** *suspicion*, pointing & representing. **(C)** stirring, acting & embodying.

We coded a response as **sequential-only when two or more meaningful gesture units were produced in temporal succession and no simultaneous pairing was present.** Sequential combination was therefore defined by the ordering of representational units within a response (Figure 3). Successive movements were treated as separate units only when they contributed distinguishable semantic information. Repetition of the same movement without additional semantic content remained part of a single unit. Sequential relations were recorded using the symbol “+.” For door, the participant first traced its outline and then mimicked knocking on it (drawing + acting, Figures 3A,B). Similarly, to depict *intelligence*, the participant first pointed to the head and then produced a thumbs-up gesture (pointing + symbolic mapping, Figures 3C,D).

**Figure 3:**
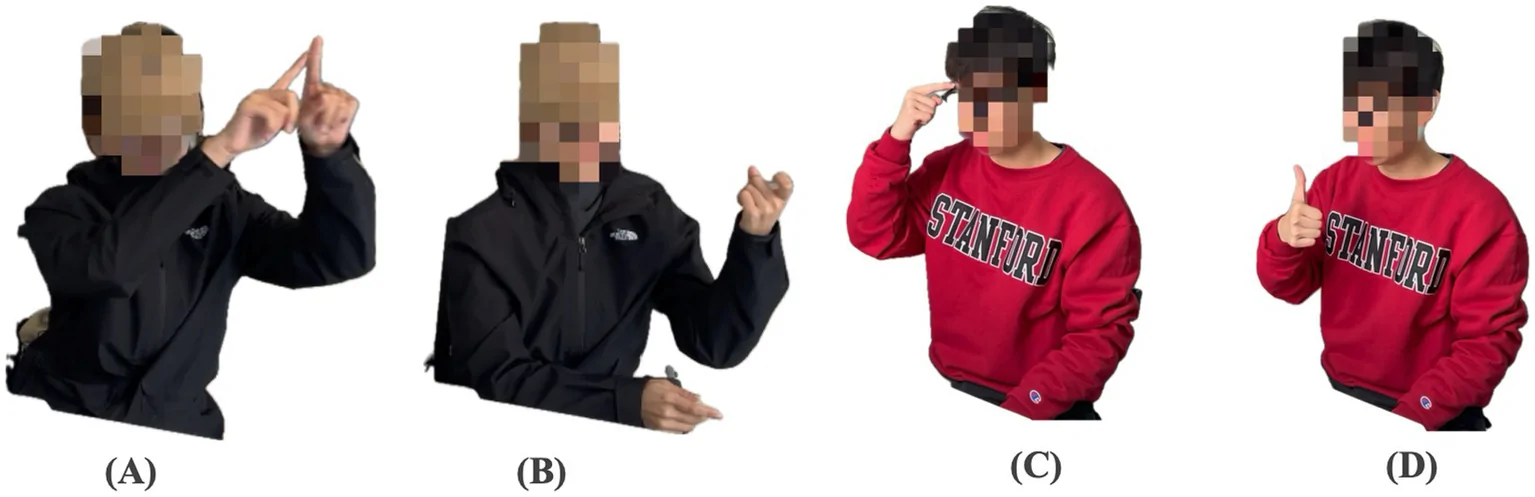
Examples of sequential-only combinations. **(A,B)** *door*, drawing + acting. **(C,D)** *intelligence*, pointing + symbolic mapping.

A response was coded as **mixed** when it contained both sequential and simultaneous organization. At least one simultaneous bimanual pairing occurred within a larger sequential progression. For instance, in the gesture for *nail*, one hand first embodied the nail by holding the index finger upright, while the other hand pointed to it. In the following part of the response, the embodying hand remained in place, with only a slight inward adjustment, and the other hand changed to an acting movement depicting hammering. This response was therefore coded as mixed because it consisted of two successive simultaneous pairings, pointing & embodying followed by acting & embodying (Figure 4).

**Figure 4:**
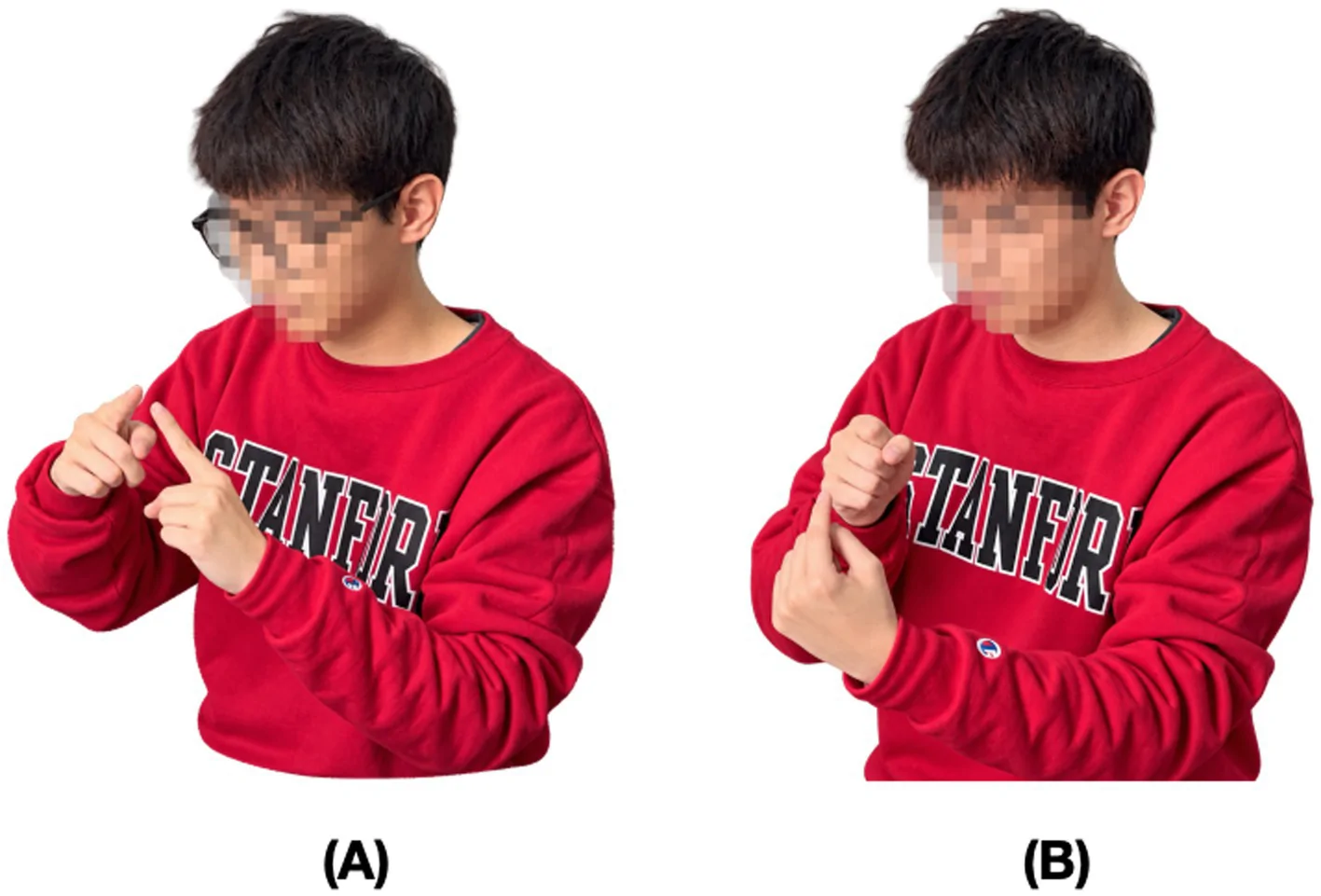
Example of a mixed combination for *nail*. **(A)** Pointing & embodying. **(B)** Acting & embodying. The response was coded as mixed because the simultaneous pairing changed across successive temporal stages.

#### 3.4.4 Coding reliability

To assess coding reliability, a second coder, a Ph.D. student specializing in linguistics, coded 20% of the dataset (*N* = 1,870 responses). The second coder was blind to the research purpose and the first author’s annotations. We assessed agreement for the iconic strategy assigned to each meaningful gesture unit and for the classification of each response as non-combinatorial, sequential-only, simultaneous-only, or mixed. Agreement was 88% for the coding of iconic strategies (Cohen’s κ = 0.820) and 91% for the classification of responses by organizational format (Cohen’s κ = 0.850).

The assignment of focal and anchoring roles in simultaneous pairings was assessed separately. A third coder with two years of experience in gesture research independently coded 40% of the simultaneous data, with 98% agreement in the assignment of focal and anchoring roles (Cohen’s κ = 0.973). Disagreements in all coding dimensions were resolved through discussion before the final analyses were conducted.

### 3.5 Preparation of datasets for analysis

The full dataset contained 9,348 trials, corresponding to 38 participants responding to 246 target concepts. Responses were first screened at the response level. Trials in which participants did not produce a gesture for the target concept were coded as pass responses (*n* = 1,606). Responses that involved only facial expressions or whole-body movements without observable manual involvement were coded as non-manual-only responses (*n* = 158). The remaining 7,584 silent gesture responses contained at least one meaningful manual unit and formed the dataset for the main analyses of iconic strategies and their combinations. Non-manual-only responses were examined separately in a supplementary analysis.

Applying the coding described in Section 3.4.3, 1,939 of the 7,584 silent gesture responses were combinatorial, including 1,242 sequential-only responses, 585 simultaneous-only responses, and 112 mixed responses. The remaining 5,645 responses were non-combinatorial. These responses were used to examine whether more than one meaningful gesture unit occurred and how the units were organized. At this level, each response contributed one observation, with mixed responses retained as a separate category.

For the analyses of simultaneous organization, we extracted all bimanual pairings from simultaneous-only and mixed responses. After excluding one pairing containing a unit coded as other, the dataset included 696 responses with at least one valid simultaneous pairing, yielding 708 valid bimanual pairs. Each pairing contained one unit from the focal hand and one from the anchoring hand, yielding 1,416 gesture units in total. The pairings were analyzed in terms of both iconic strategies and broader iconicity types.

For the analysis of sequential organization, we extracted the temporal progression from sequential-only and mixed responses. After excluding units coded as other, the sequential analysis contained 1,343 responses with a sequential component, yielding 2,790 identifiable gesture units. Each gesture unit was assigned a relative position according to its order within the response. The first and last units were coded as initial and final, respectively, while intervening units were coded as medial. These labels referred only to relative position within the response and did not assign a grammatical or discourse function to the unit.

Two further analysis units were derived from the sequential strings. First, unordered co-occurrence pairs were extracted to identify which iconic strategies were selected within the same sequential response, regardless of order. For two-unit sequences, the two units formed one co-occurrence pair. For sequences with three or more units, all possible pairwise combinations were extracted. Co-occurrence pairs were used to identify recurrent strategy pairings before examining whether those pairings also showed preferred temporal orders. Second, adjacent transitions were extracted to examine how iconic strategies unfolded over time. For a two-unit sequence, the transition was the ordered relation between the first and second unit. For a sequence A + B + C, the adjacent transitions were A → B and B → C. Transition coding captured local temporal progression while avoiding the sparsity that would result from treating each full multi-unit sequence as a separate pattern.

Mixed responses contributed to the structural datasets according to the organization being analyzed. At the response level, mixed responses were retained as a separate organizational type, allowing them to be distinguished from sequential-only and simultaneous-only responses. For the analysis of simultaneous organization, each valid bimanual pairing was retained in full. For the sequential analysis, each simultaneous interval was represented by its focal-hand unit, yielding one unit for each temporal step in the progression. The corresponding anchoring-hand unit remained represented in the simultaneous dataset. This treatment preserved a single ordinal progression for the analyses of position and transition while retaining the complete bimanual structure for the analyses of simultaneous organization. Detailed rules for classifying combination structure and deriving the structural variables are provided in Sections 5 and 6 of the Supplementary Codebook.

### 3.6 Statistical analysis

All analyses were conducted in R (R Core Team, 2025). Because responses were repeatedly obtained from the same participants and for the same items, binomial generalized linear mixed-effects models (GLMMs) were used for the analyses at the level of the response. Models were fitted with the *glmmTMB* package (Brooks et al., 2017), and estimated probabilities were obtained with *emmeans* (Lenth and Piaskowski, 2025). Before examining combinatorial responses, we first tested whether the occurrence of pass responses and manual silent gesture responses varied with concreteness or conceptual category across all 9,348 trials. Non-manual-only responses were relatively infrequent and were therefore examined separately, with their relation to concreteness reported in a supplementary analysis.

The overall model compared all combinatorial responses (*n* = 1,939) with non-combinatorial responses (*n* = 5,645), using all 7,584 manual responses. Format-specific models compared sequential-only (*n* = 1,242), simultaneous-only (*n* = 585), or mixed responses (*n* = 112) with the same 5,645 non-combinatorial responses, yielding sample sizes of 6,887, 6,230, and 5,757, respectively (Supplementary Material S2). Format-specific probabilities are conditional on inclusion in the corresponding binary comparison. We tested concreteness and conceptual category in separate models. The general model for concreteness was: Combinatorial response ~ Concreteness + (1 + Concreteness | Participant) + (1 | Item). Concreteness was treatment-coded with abstract as the reference level. The general model for conceptual category was: Combinatorial response ~ Conceptual category + (1 | Participant) + (1 | Item) + (1 | Participant × Conceptual category). For the random slope by participant, the contrast was centered at −0.5 for abstract concepts and 0.5 for concrete concepts. Conceptual category was treatment-coded with actions with objects as the reference category. Random intercepts for participant and item were included in all models, while a random slope for concreteness by participant was included when it could be estimated reliably. For conceptual category, variation across participants was represented by a participant-by-category random component. When the fuller structure could not be estimated reliably, random intercepts for participant and item were retained. The contribution of each fixed effect was assessed with a likelihood-ratio test (LRT), for which the full model was compared with a reduced model retaining the same structure for the random effects. All reported models converged successfully, with positive-definite Hessians and no boundary estimates. Complete formulas, final random-effects structures, and model diagnostics are provided in Supplementary Material S13.

For simultaneous combinations, the distribution of strategies across focal and anchoring roles was summarized descriptively. We then examined whether iconicity types and iconic strategies formed recurrent pairings across the hands. Pearson chi-square statistics and Cramér’s V were used to summarize the overall associations, while standardized residuals identified the pairings that contributed most strongly to the observed distributions. For simultaneous associations, anchoring-hand type or strategy labels were shuffled within participants and, in a separate test, within concepts, with focal-hand labels held fixed. Each procedure retained the number of pairings and the marginal frequencies of both labels within the relevant stratum. For category associations, category labels were shuffled across concepts within concrete and abstract strata, with all pairings from each concept kept together. Each test used 5,000 permutations. The participant- and concept-stratified tests provided complementary checks, with agreement between them supporting the overall association (Supplementary Material S15).

For sequential combinations, we examined the position of gesture units, the co-occurrence of iconic strategies, and transitions between adjacent gesture units. Initial and final positions were compared using conditional logistic regression, with each response treated as a matched stratum and drawing serving as the reference strategy. Medial units were excluded from this comparison. The model was specified as Final position ~ Iconic strategy + strata(Response). To account for repeated observations, standard errors were estimated using HC1 two-way cluster-robust covariance by participant and concept (Zeileis et al., 2020). Unordered co-occurrence pairs were used to identify recurrent strategy pairings, while adjacent transitions were used to examine how iconic strategies were ordered over time. The overall association between strategies in adjacent positions was summarized using Pearson’s chi-square statistic and Cramér’s V. The same two stratified permutation procedures were applied by shuffling the second strategy within participants or within concepts while holding the first strategy fixed (5,000 permutations per test, Supplementary Material S15). Directionality was then examined for pairs of different iconic strategies that occurred in both directions and contributed at least 10 adjacent transitions. The 26 eligible pairs were analyzed jointly using a binomial generalized linear mixed-effects model with crossed random intercepts for participant and concept, fitted with the *lme4* package (Bates et al., 2015). For each pair, the observed majority direction was coded as 1 and the reverse direction as 0. The variable labelled Preferred direction in the model records this observed majority. Each pair-specific coefficient was evaluated using a two-sided Wald z test against zero log odds, equivalent to a conditional probability of 0.50. To estimate the directional preference for each pair directly, the model was specified without an intercept as Preferred direction ~ 0 + Pair + (1 | Participant) + (1 | Concept). The Benjamini-Hochberg procedure was applied separately to the ten comparisons with drawing in the position analysis and to the 26 tests of directional preference. An observed majority was interpreted as a statistically supported directional preference only when its two-sided test remained significant after correction across the 26 pairs. Detailed results are provided for directionality, including counts in both directions, model estimates, 95% CIs, and raw and BH-adjusted *p*-values (Supplementary Material S10), initial–final position comparisons (Supplementary Material S14), and permutation tests and sensitivity analyses (Supplementary Material S15).

## 4 Results

### 4.1 Distribution of iconic strategy combinations across concepts

Before analyzing the combinatorial responses, we first examined whether pass responses and silent gesture responses varied with concreteness and conceptual category across all trials. Pass responses were more frequent for abstract concepts (28.63%) than for concrete concepts (11.24%), and they were related to both concreteness (*χ*^2^(1) = 40.86, *p* < 0.001) and conceptual category (*χ*^2^(11) = 166.22, *p* < 0.001). The highest pass rates were found for social concepts (46.32%), attributes (41.01%), and social role entities (30.79%). The elicitation of silent gesture responses also varied with both concreteness (*χ*^2^(1) = 42.42, *p* < 0.001) and conceptual category (*χ*^2^(11) = 141.72, *p* < 0.001). Non-manual-only responses were rare overall (1.69% of all trials) and occurred most frequently for emotions (8.25%) and actions without objects (7.64%). Further details are provided in Supplementary Material S12.

Of the 7,584 silent gesture responses, 1,939 contained more than one meaningful iconic gesture unit. Sequential-only combinations accounted for 1,242 responses (16.38% of manual responses; 64.05% of combinatorial responses), simultaneous-only combinations for 585 responses (7.71% of manual responses; 30.17% of combinatorial responses), and mixed combinations for 112 responses (1.48% of manual responses; 5.78% of combinatorial responses). The remaining 5,645 silent gesture responses (74.43%) were non-combinatorial. The associations of concreteness and conceptual category with combinatorial response structure are summarized in Figure 5.

**Figure 5:**
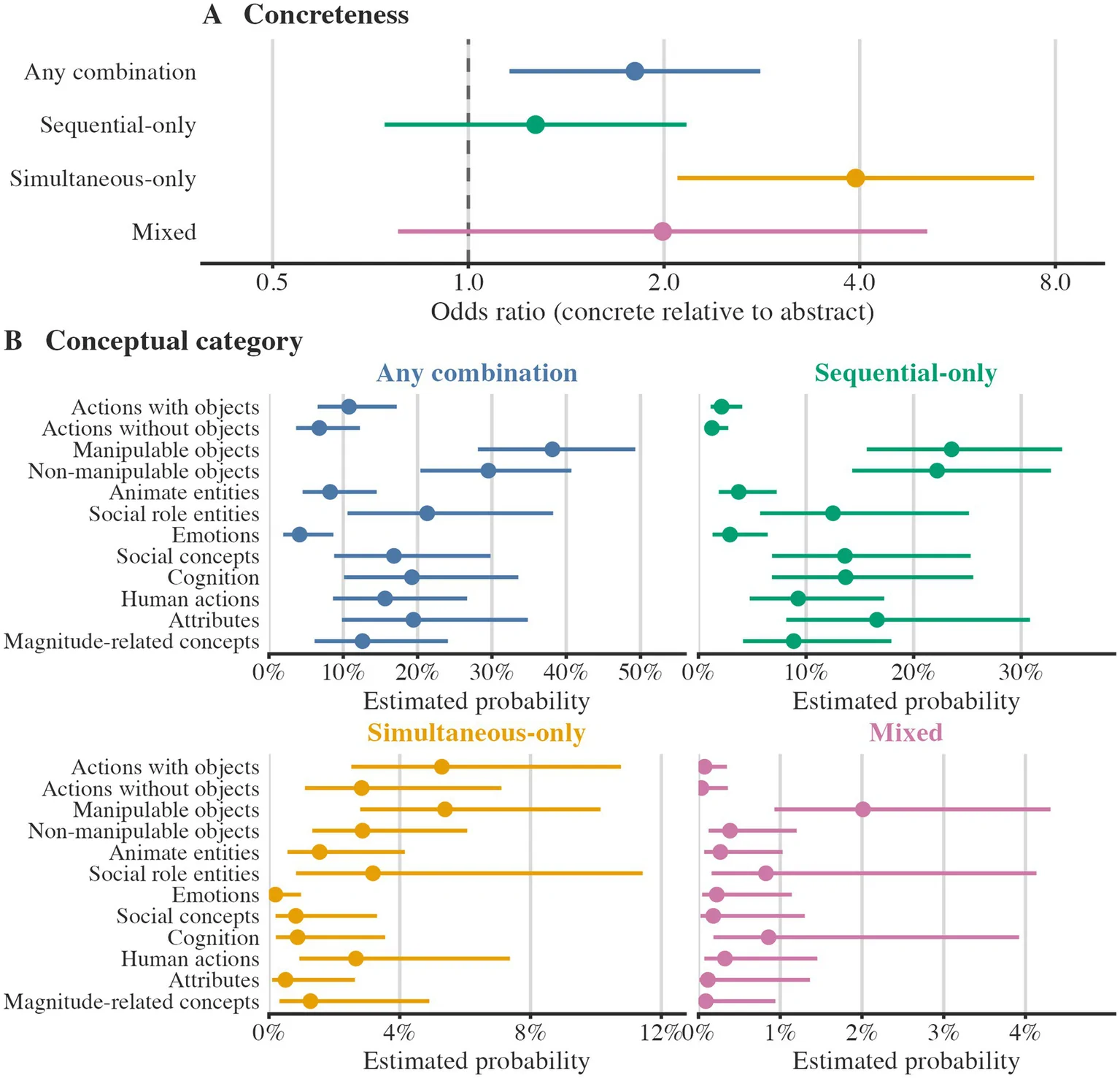
Associations of concreteness and conceptual category with combinatorial response structure. **(A)** Odds ratios for concrete relative to abstract concepts. **(B)** Estimated probabilities by conceptual category. Points show model estimates and horizontal lines indicate 95% confidence intervals. The overall comparison includes all manual responses (*N* = 7,584). Format-specific comparisons include only the named format and non-combinatorial responses, with *N* = 6,887 for sequential-only, *N* = 6,230 for simultaneous-only, and *N* = 5,757 for mixed responses.

We first examined whether the occurrence of combinatorial responses varied with concreteness or conceptual category. Concreteness was related to the occurrence of combinatorial responses (*χ*^2^(1) = 6.85, *p* = 0.009). Combinatorial responses were more likely for concrete than for abstract concepts (OR = 1.80, 95% CI [1.16, 2.81]). Conceptual category was also related to the occurrence of combinatorial responses (*χ*^2^(11) = 73.68, *p* < 0.001). Estimated probabilities were highest for manipulable objects and non-manipulable objects, followed by social role entities, attributes, and cognition concepts, and were lowest for emotions, actions without objects, and animate entities.

We next examined sequential-only, simultaneous-only, and mixed responses in separate comparisons with non-combinatorial responses. The estimated probability of each format refers to its corresponding binary comparison. Concreteness was not related to sequential-only responses (*χ*^2^(1) = 0.77, *p* = 0.380) or mixed responses (*χ*^2^(1) = 2.13, *p* = 0.144). It was, however, related to simultaneous-only responses (*χ*^2^(1) = 18.73, *p* < 0.001), which were more likely for concrete than for abstract concepts (OR = 3.94, 95% CI [2.10, 7.42]). Conceptual category, by contrast, was related to the occurrence of all three organizational formats. For sequential-only responses, the association with conceptual category was significant (*χ*^2^(11) = 122.91, *p* < 0.001). The highest estimated probabilities were found for manipulable objects, non-manipulable objects, attributes, cognition concepts, and social concepts, whereas actions without objects, actions with objects, emotions, and animate entities showed the lowest probabilities. For simultaneous-only combinations, conceptual category was also significantly associated with their occurrence (*χ*^2^(11) = 33.50, *p* < 0.001). Estimated probabilities were highest for manipulable objects and actions with objects, followed by social role entities, non-manipulable objects, and actions without objects, and were lowest for emotions, attributes, social concepts, and cognition concepts.

Mixed combinations were rare overall, but their occurrence still varied by conceptual category (*χ*^2^(11) = 39.61, *p* < 0.001). The highest estimated probability was found for manipulable objects, followed by cognition concepts, social role entities, and non-manipulable objects. Given the small number of mixed responses (*N* = 112), these category-level differences should be interpreted cautiously.

Overall, concreteness was related to the occurrence of combinatorial responses and, among the three organizational formats, to simultaneous-only responses. Conceptual category was associated with the occurrence of combinations overall and with each organizational format. Conceptual categories related to objects, especially manipulable and non-manipulable objects, were most strongly associated with the combination of iconic strategies. Sequential combinations were more broadly distributed across object and abstract categories, simultaneous combinations were concentrated in more directly depictable object- and action-related categories, and mixed combinations remained infrequent but clustered in a limited set of concept types (see Supplementary S2 for model tests and estimates and S3 for estimated probabilities).

### 4.2 Simultaneous combinations: distribution of iconic strategies across the hands and bimanual pairing

After excluding one pairing that contained a unit coded as other, the analysis included 708 valid bimanual pairings from 696 responses, yielding 1,416 meaningful gesture units. Because some responses contained more than one simultaneous pairing, the number of pairings was slightly larger than the number of responses.

We found that action-based gesture units occurred predominantly in the focal hand (322 focal-hand units vs. 9 anchoring-hand units), whereas relation-based units were concentrated in the anchoring hand (46 focal-hand units vs. 325 anchoring-hand units). Form-based units occurred frequently in both hands, with 335 focal-hand units and 373 anchoring-hand units. At the level of individual iconic strategies, acting (*N* = 320) and representing (*N* = 264) occurred most often in the focal hand, whereas embodying (*N* = 341) and locating (*N* = 291) occurred most often in the anchoring hand.

We also found that the two hands formed systematic patterns of simultaneous pairings. At the level of iconicity type, the two hands were significantly associated (*χ*^2^(12) = 373.98, *p* < 0.001, Cramér’s *V* = 0.420). Two combinations accounted for most simultaneous pairings: an action-based unit in the focal hand with a form-based unit in the anchoring hand (*N* = 295, 41.67%), and a form-based unit in the focal hand with a relation-based unit in the anchoring hand (*N* = 270, 38.14%). Pairings in which both units were form-based were the next most frequent, although they occurred much less often (*N* = 56, 7.91%). Standardized residuals showed that the first two combinations were the most strongly overrepresented (Figure 6A). We observed a similar association at the level of individual iconic strategies (*χ*^2^(72) = 745.97, *p* < 0.001, Cramér’s *V* = 0.363). The most frequent pairing was acting in the focal hand and embodying in the anchoring hand (*N* = 289, 40.82%), followed by representing and locating (*N* = 211, 29.80%). Less frequent pairings included representing and embodying (*N* = 39, 5.51%), embodying and locating (*N* = 32, 4.52%), and acting and locating (*N* = 19, 2.68%) (Figure 6B).

**Figure 6:**
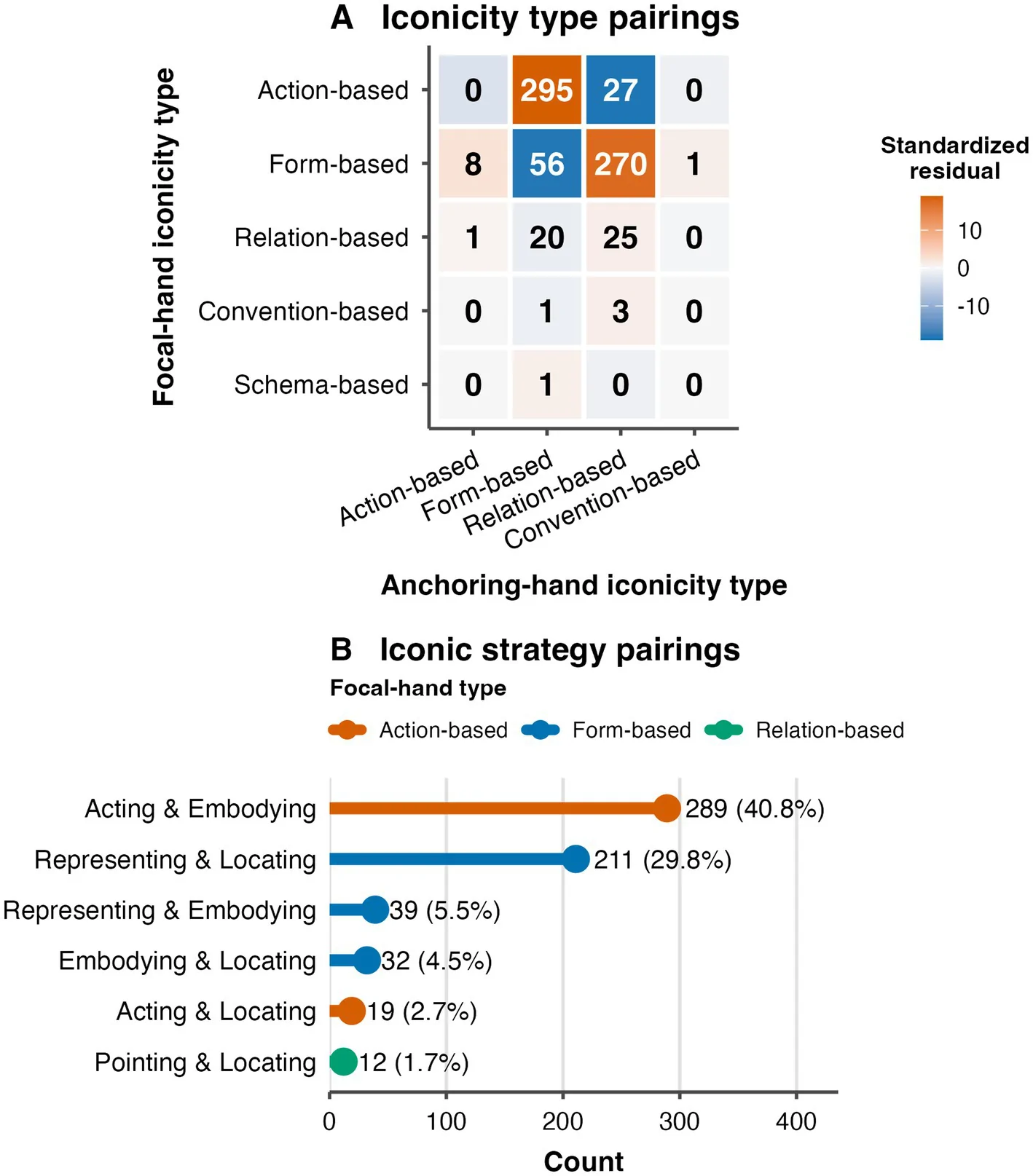
Simultaneous pairings of iconicity types and the six most frequent iconic-strategy pairings.

The distribution of bimanual pairings at the level of iconicity type also varied across conceptual categories (*χ*^2^(121) = 919.25, *p* = 0.013, Cramér’s *V* = 0.344). For actions with objects and manipulable objects, the most frequent pairing was an action-based unit in the focal hand with a form-based unit in the anchoring hand, accounting for 115 of 151 pairs (76.16%) and 155 of 247 pairs (62.75%), respectively. By contrast, for actions without objects, animate entities, non-manipulable objects, and cognition concepts, a form-based unit in the focal hand was most often paired with a relation-based unit in the anchoring hand, accounting for 78 of 79 pairs (98.73%), 34 of 36 pairs (94.44%), 54 of 78 pairs (69.23%), and 16 of 24 pairs (66.67%), respectively. For magnitude-related concepts, pairings of two relation-based units (7 of 18 pairs, 38.89%) and pairings of a relation-based unit in the focal hand with a form-based unit in the anchoring hand (6 of 18 pairs, 33.33%) together accounted for most simultaneous pairings. Descriptive distributions and standardized residuals are provided in Supplementary Materials S4–S6. Results of the permutation tests are provided in Supplementary Material S15.

### 4.3 Sequential combinations: positional organization and directional transitions

After excluding units coded as other, the analyses included 1,343 responses with a sequential component, yielding 2,790 gesture units, 1,553 unordered co-occurrence pairs, and 1,447 adjacent transitions. Most of these responses were short: 1,241 contained two sequential units (92.41%), 100 contained three units (7.45%), and only two contained four units (0.15%).

We found that iconic strategies differed systematically between initial and final positions within sequential combinations (*χ*^2^(10) = 107.58, *p* < 0.001). Of the occurrences of drawing in either initial or final position, 505 of 614 (82.25%) were in initial position, and the corresponding proportion for embodying was 198 of 276 (71.74%). The opposite pattern was observed for acting, path mapping, and symbolic mapping: 488 of 691 occurrences of acting (70.62%), 65 of 77 occurrences of path mapping (84.42%), and 176 of 215 occurrences of symbolic mapping (81.86%) were in final position.

Among the 1,553 unordered co-occurrence pairs, acting with drawing was the most frequent (*N* = 323, 20.80%), followed by acting with embodying (*N* = 133, 8.56%) and pairs in which both units were coded as acting (*N* = 118, 7.60%). Other frequent pairs included pointing with symbolic mapping (*N* = 82, 5.28%), drawing with representing (*N* = 78, 5.02%), drawing with embodying (*N* = 72, 4.64%), and drawing with pointing (*N* = 63, 4.06%). These distributions indicate that action-based information was often combined with strategies that introduced form, entity, or relational information.

When temporal order was taken into account, iconic strategies in adjacent units showed a significant association (*χ*^2^(100) = 1130.69, Cramér’s *V* = 0.280), which remained significant in cluster-stratified permutation tests conducted within participants (*p* < 0.001) and within concepts (*p* = 0.043). The most frequent transition was drawing to acting (*N* = 257, 17.76%), followed by embodying to acting (*N* = 114, 7.88%), acting to acting (*N* = 113, 7.81%), pointing to symbolic mapping (*N* = 67, 4.63%), and drawing to representing (*N* = 66, 4.56%). Drawing also preceded embodying (*N* = 49, 3.39%) and pointing (*N* = 43, 2.97%) frequently, while schema mapping frequently preceded symbolic mapping (*N* = 40, 2.76%).

Of the 26 recurrent pairs examined for directionality, 14 showed a preferred temporal order after Benjamini–Hochberg correction. Drawing occurred before acting more often than in the reverse order (257/301, 85.38%, *p* < 0.001) and likewise preceded representing (66/73, 90.41%, *p* < 0.001). Similarly, embodying also preceded acting in most occurrences of this pair (114/124, 91.94%, *p* < 0.001). In addition, for sequential pairs involving symbolic mapping, both pointing (67/77, 87.01%, *p* < 0.001) and schema mapping (40/44, 90.91%, *p* < 0.001) occurred more often before symbolic mapping than in the reverse order.

It is important to note that because most sequential responses contained only two units, the frequencies of full sequences closely paralleled those of the adjacent transitions. The most frequent full sequences reflected the same organization. The two most common sequences were drawing followed by acting (*N* = 232, 17.27%) and embodying followed by acting (*N* = 103, 7.67%). Acting followed by acting was also frequent (*N* = 84, 6.25%), as were drawing followed by representing (*N* = 64, 4.77%) and pointing followed by symbolic mapping (*N* = 61, 4.54%). Distributions of sequential positions, unordered co-occurrences, adjacent transitions, and directional preferences are provided in Supplementary Materials S7–S10. Results of the matched comparison of initial and final positions and the sensitivity analyses are provided in Supplementary Materials S14, S15.

### 4.4 Mixed combinations

Mixed combinations were infrequent, accounting for 112 of the 1,939 combinatorial responses (5.78%) and occurring for 61 concepts across all 12 conceptual categories. They were most numerous for manipulable objects (*N* = 56, 8.62% of combinatorial responses in this category), followed by cognition concepts (*N* = 15, 15.63%) and non-manipulable objects (*N* = 12, 3.16%). Although mixed combinations were infrequent, their distribution varied considerably across concepts. A small number of concepts accounted for a disproportionate share of mixed responses. For example, *contrast* (cognition) elicited mixed combinations in 12 instances, followed by *shoes* (manipulable object; 10 instances) and *nail* (manipulable object; 9 instances). Some concepts also showed recurring arrangements of sequential and simultaneous components. For *contrast*, the most frequent pattern was representing + representing & locating, in which an initial gesture established a comparative frame, followed by a simultaneous combination that integrated relational information with spatial anchoring. Similarly, for manipulable objects such as *shoe* and *nail*, the patterns of mixed combinatorial responses typically involved a sequential progression paired with simultaneous action–object or spatial integration (e.g., embodying + acting & embodying, representing & locating + pointing). Distributions of mixed responses by concept and conceptual category, including the most frequent patterns for each concept, are provided in Supplementary Material S11.

Although mixed responses were infrequent, they provide a small but clear set of cases in which sequential and simultaneous organization occurred within the same response. Mixed combinations tended to occur for concepts that invited both entity or relational grounding and sequential elaboration of action, function, or comparison. Given the relatively small number of mixed responses, we focus here on the patterns that were observed and do not subject their internal structures to further inferential analyses.

## 5 Discussion

Research on silent gesture has revealed that hearing non-signers do not gesture randomly when speech is removed. Even without an established communicative system, they produce systematic iconic form-meaning mappings, and these mappings are shaped by semantic category (Flaherty and Schouwstra, 2025; Ortega et al., 2019; Ortega and Özyürek, 2020a; Özçalışkan et al., 2016; Pouw et al., 2021). Ortega and Özyürek (2020b) further showed that the representation of a concept can involve combinations of different iconic strategies, particularly for object concepts, thereby extending the analysis of iconicity from individual gestures to combinations within a larger gestural expression. What remains less clear, however, is how the meaningful gesture units that constitute such combinations are internally organized. Do recurrent positional and directional relations emerge when these units unfold sequentially, and do systematic bimanual pairings emerge when distinguishable information is distributed simultaneously across the two hands? How, in turn, are sequential and simultaneous relations brought together within mixed combinations? The present study addressed these questions by extending the analysis of combinatorial structure from the presence and co-occurrence of iconic strategies to the relations among meaningful gesture units, while also broadening the conceptual range from the predominantly concrete domains examined in earlier work to a substantially wider set of concrete and abstract concepts. We found that combinations accounted for a minority of silent gesture productions, with their occurrence related to both concreteness and conceptual category. More importantly, the combinations displayed recurrent internal organization, with systematic positional and directional relations in sequential combinations, recurrent pairings across the two hands in simultaneous combinations, and a smaller set of mixed combinations in which the two forms of organization occurred within the same expression.

We anticipated that concrete concepts, those with visible shapes, perceptual availability, and sensorimotor experience, would elicit more combinations. The reasoning is straightforward. The manual modality is particularly well-suited to visible shape, bodily action, object manipulation, movement, and spatial relations (Dingemanse et al., 2015; Hostetter and Alibali, 2008, 2019; Mamus et al., 2025; Perniss et al., 2010; Perniss and Vigliocco, 2014; Zlatev et al., 2020), all of which tend to contribute more strongly to concrete conceptual representations, although both concrete and abstract concepts draw on multiple experiential sources (Altarriba et al., 1999; Altarriba and Bauer, 2004; Conca et al., 2021; Crutch and Warrington, 2005; Villani et al., 2019). If combinatorial production is driven by the amount of depictable material a concept affords, concrete concepts should have elicited more combinations overall. Against this background, we found that concrete concepts were more likely than abstract concepts to elicit combinations of meaningful gesture units. One possibility is that experiential dimensions receiving relatively greater weight in many concrete concepts also align closely with the visuospatial and motoric resources available for gestural depiction, allowing more than one aspect of the concept to receive a distinct manual realization within the same expression (Mamus et al., 2025; Ortega and Özyürek, 2020b; Villani et al., 2019).

The broad relation with concreteness, however, did not extend equally to different forms of combinatorial organization. Concreteness was related to simultaneous-only combinations, which occurred more frequently for concrete concepts, whereas no reliable relation emerged for sequential-only or mixed combinations. One possible explanation behind this pattern is that simultaneous structuring allows gesture producers to keep related aspects of a meaning visible within the same temporal window (Slonimska et al., 2020, 2022). This possibility is especially relevant to concrete concepts, which often involve separable yet interdependent entities, actions, and spatial relations that can be depicted on a surface, against a ground, along a path, or within a spatial setting. Bradley and Wilbur (2023), for example, found that two-handed silent gestures were more frequent for transitive than for intransitive events, and in many productions the two hands could be analyzed as mapping onto different event participants. In their example of cutting *bread*, one hand represented the bread and the other depicted the action performed with the knife.

Why, then, did the same broad distinction fail to differentiate sequential-only combinations? One possible explanation is that the need to distribute meaning across successive, independently meaningful gestures is less closely tied to concreteness than the availability of information that can be mapped directly onto the manual-visual modality. Concrete concepts tend to rely more strongly on perceptual and sensorimotor experience, and such information is particularly amenable to analogue depiction through the hands and body (Mamus et al., 2025; Villani et al., 2021). Yet this advantage concerns the accessibility of material for gestural depiction. It does not in itself specify whether the selected information will be realized in one gesture or separated into several gestures unfolding over time. Research on newly created gestural systems shows that the use of compositional sequences increases when the meaning to be conveyed contains components or relations that benefit from separate expression, suggesting that serial organization is sensitive to how information can be decomposed and related, rather than simply to how directly it can be depicted (Bohn et al., 2019). Our findings further suggest that the possibility of such segmentation is not confined to perceptually rich meanings. Abstract concepts provide fewer directly visible properties, yet they may draw on heterogeneous experiential sources, including social, affective, interoceptive, linguistic, and embodied information (Recchia and Jones, 2012; Banks and Connell, 2023). Once a concept has yielded more than one gesturally realizable component, arranging those components successively may therefore depend more on their separability and relational organization than on whether the target falls on the concrete or abstract side of the broader distinction. This would explain why the sensorimotor advantage associated with concrete concepts was visible in combinatorial production overall, and particularly in simultaneous organization, without producing a corresponding increase in sequential-only combinations. Our findings are consistent with Schiefner (2025), who found that abstract concepts elicited more pass responses but that concreteness was unrelated to the number of gestures once a manual response was produced. Similarly, Ortega and Özyürek (2020b) found substantial differences in gesture number and strategy combinations among action and object concepts that were all concrete, indicating that variation in multiple gesture production can arise from conceptual properties that are not captured by concreteness.

Conceptual category revealed variation not captured by the binary concreteness distinction. One way of understanding this variation is to consider the different kinds of information that have been shown to enter manual representation across conceptual categories, including characteristic actions, object form, bodily configuration, and spatial relations (Hwang et al., 2017; Masson-Carro et al., 2016; Ortega and Özyürek, 2020a, 2020b; van Nispen et al., 2017). We found an informative contrast between manipulable objects and actions with objects, both of which involve characteristic actions through grasping, use and manipulation, while their representation also draws on perceptual information about visible and structural properties such as shape and configuration (Kellenbach et al., 2003). Previous studies have shown that they are frequently represented through handling or acting, while form-based techniques remain available as alternative or additional routes of depiction (Masson-Carro et al., 2016; Ortega and Özyürek, 2020a, 2020b; van Nispen et al., 2017). Here, we found that manipulable objects were among the categories most likely to recruit multiple meaningful gesture units, with both sequential and simultaneous organization occurring prominently in this category. Our findings further reveal that the iconic resources previously documented for object representation can be coordinated in different ways once several meaningful units enter the same gestural expression. In sequential responses, object form and characteristic use could be expressed successively, consistent with patterns previously reported for object representation (Ortega and Özyürek, 2020b; van Nispen et al., 2017). In simultaneous combinations, one hand could represent the object while the other hand enacted a handling action, keeping object and use perceptually co-present. Actions with objects, however, showed a different organizational profile. We found that actions with objects were among the categories in which simultaneous-only combinations occurred most frequently, whereas sequential-only combinations remained comparatively uncommon. This concentration in simultaneous organization is consistent with Bradley and Wilbur’s (2023) finding that two-handed silent gestures were more frequent for transitive events, in which the two hands could map onto different event participants. In terms of the successive arrangement of iconic gesture units, the action-object relation represented within a single bimanual configuration may reduce the need for successive elaboration, which was more frequent for manipulable objects.

Consistent with previous findings (Hwang et al., 2017; van Nispen et al., 2017; Mamus et al., 2025), we found that non-manipulable objects were expressed systematically through drawing and other form-based strategies. Yet these concepts also elicited combinations involving spatial relations, as shown by the frequent pairing of form-based and relation-based units. An entity can be depicted in relation to a ground or surface, providing a basis for combining gestures even when manipulation is not central to the target meaning (Mittelberg, 2019). We also found that attributes, social concepts, and cognition concepts were among the abstract categories most strongly associated with sequential-only combinations, while simultaneous-only combinations were uncommon. For attributes, a carrier, scale, or reference point may provide a basis for depicting the relevant property (Langacker, 1993, 2008). Similarly, a social concept may be depicted through participants and their relation within an interaction frame (Barsalou, 2005; Fillmore, 1982). For cognition concepts, spatial locations, image schemas, comparisons, and relational mappings may likewise provide resources for manual representation (Johnson, 1987; Gentner and Markman, 1997). In all these cases, sequential organization allows separate externalization of different representational dimensions across successive gesture units.

Besides, we found that social concepts and attributes also showed some of the highest rates of pass responses, a pattern shared with social role entities. This convergence is informative, as all three categories depend strongly on socially learned relations, roles, and patterns of behavior. Large-scale analyses of semantic knowledge show that social information forms a distinct dimension of word meaning and is particularly associated with auditory experience and actions involving the mouth and head, consistent with an important role of verbal interpersonal interaction in the representation of social meaning (Diveica et al., 2024). It has also been shown that concepts referring to social traits, including meanings such as *kind*, *sincere*, and *loyal*, draw on simulations of socially interpretable behavior as well as information derived from language (Alcalá-López et al., 2024). Under the present task, such meanings may provide fewer direct mappings onto a single manual form, which may account for their relatively frequent pass responses. When participants found a manual route, however, different elements of the selected social situation could be introduced successively, allowing a referent, behavior, relation, or evaluation to contribute separate components to the expression.

At the other end of the distribution, emotions, actions without objects, and animate entities showed comparatively low rates of combination. Emotion concepts can draw on facial expression, bodily stance, or affective display, so part of their communicative force may lie outside the manual units analyzed here (Borghi et al., 2014; Keränen, 2021). Actions without objects can often be enacted directly because there is no separate affected object that needs to be represented (Zacks and Tversky, 2001). The low rate of manual combinations and the relatively frequent non-manual-only responses for emotions are consistent with the contribution of facial and bodily cues to emotion perception (Heesen et al., 2024). Pantomimic action can also involve movements beyond the hands (Placiński et al., 2023). Animate entities, similarly, often invite personification, allowing the body to stand for the referent without further decomposition (Hwang et al., 2017; Mamus et al., 2025). These differences help explain why the analysis of conceptual categories revealed variation in combinations beyond that captured by the broad distinction between concrete and abstract concepts.

Beyond the question of which concepts elicit combinations, our data also reveal how these combinations are structured internally. Simultaneous combinations of iconic strategies showed recurrent patterns in how different representational components were coordinated across the two hands. The two commonest pairings observed in our research, namely, acting with embodying and representing with locating, illustrate different representational relations within simultaneous combinations of iconic strategies. The manual-visual modality allows different articulators to carry distinct semantic components within the same temporal window (Emmorey, 2002; Vermeerbergen et al., 2007; Meier, 2012; Brentari, 2019). Slonimska et al. (2022) found that established sign languages can use simultaneity to keep related meaning components close together. Our data further reveal that hearing non-signers also recruited it selectively, especially when conceptual components could be meaningfully distributed across the hands. Gestures can profile routinized aspects of embodied experience, so that a partial action or object cue gives access to a larger frame (Mittelberg, 2019; Mittelberg and Waugh, 2014). When depicting manipulable objects and actions with objects, simultaneity allows action and object to remain perceptually co-present. One interpretation is that the pairing draws on a familiar relation between an action and the object involved, consistent with accounts of how embodied experience informs gestural depiction (Mittelberg, 2019).

The form-based & relation-based pairing suggests a different route to simultaneous organization. In its most recurrent realization, one hand formed an inverted V-shaped handshape, representing a person or entity, while the other was held flat to represent a ground, surface, or setting. It often anchors an entity within a spatial or relational frame for non-manipulable objects that involve size, outline, location, or spatial configuration rather than manipulation; animate entities that involve a relation between an embodied representation and the space it occupies; and for cognition concepts which can be grounded through spatialized schemas (Mittelberg, 2017, 2019). This type of configuration has been repeatedly documented across research on the manual-visual modality (Cormier et al., 2013; Loos et al., 2022; Engberg-Pedersen et al., 2025). Within this gesture, a minimal bodily and spatial configuration could stand for a broader scene in which an entity was situated, contrasted, or represented in relation to its surroundings (Mittelberg, 2017, 2019). Recent work on spatial descriptions similarly shows that speakers can express relations between figure and ground by placing their hands in relation to one another, with handshape also contributing information about the represented entities (Ünal et al., 2025). From this perspective, the representing & locating pairing can be understood as a metonymic means of constructing a spatial scene. The findings suggest that this familiar figure–ground configuration can be recruited beyond the depiction of literal events. Hearing non-signers also used it to combine an entity representation with a spatial locus or setting when expressing individual concepts.

We now turn to the analysis of sequential combinations, which were the most frequent type of combinatorial response. Form-based iconic resources (i.e., drawing and embodying) were more likely to occur initially, whereas acting, path mapping, and symbolic mapping were more likely to occur later. Several recurrent strategy pairs showed clear directional preferences. For example, drawing preceded acting in 85.38% of transitions involving the two strategies, and embodying preceded acting in 91.94%. This order is unlikely to follow solely from event chronology, since an object’s form and its characteristic use have no inherent temporal sequence. One possible explanation concerns the relation between establishing a referent and depicting an action associated with it. An initial depiction of object form may establish a referent for a subsequent action, consistent with the preference for placing objects before descriptions of their properties in judgements of gesture sequences (Holtz et al., 2025). Importantly, pointing could occur in different positions within individual sequences, as illustrated by drawing followed by pointing for *center* and pointing followed by symbolic mapping for *intelligence*. In these examples, pointing could specify a location within a preceding depiction or establish a bodily reference for a subsequent evaluation, suggesting that its position may depend on the referential relation between successive units.

The predominance of sequential combinations in our data is particularly informative, given the possibility of expressing distinct components simultaneously across the two hands. Established sign languages make extensive use of such simultaneous possibilities, coordinating distinct meaning components through depicting constructions, spatial mappings, constructed action, and other structures that draw on multiple articulators (Emmorey, 2002; Perniss, 2007; Vermeerbergen et al., 2007; Loos et al., 2022; Slonimska et al., 2022). For hearing non-signers, however, Slonimska et al. (2022) found less frequent use of configurations in which one hand maintained an action while the other expressed a further action. One possible explanation concerns the demands of coordinating distinct depictions across the hands, as their handshapes, movements, and relative positions must jointly reflect the intended relation between the represented components (Gulamani et al., 2022). Producing the units successively might allow participants to reshape and reposition their hands for each depiction, which may have favored sequential combinations when several iconic strategies were used to express an individual concept. Where one hand could maintain a representation of an object while the other enacted its use, a simultaneous pairing could also be incorporated into the unfolding sequence.

Mixed combinations were rare and varied in form, although repeated instances were particularly evident for concepts such as *contrast*, *shoes*, and *nail*. Their low frequency may partly reflect the interval of four seconds available to read each word and generate a response, which may have limited opportunities to plan and produce sequences containing simultaneous pairings. Some of these combinations may have arisen as an existing depiction was extended to include an action involving its referent. For *nail*, for example, a recurrent pattern began with a depiction of the object, which was then maintained on one hand while the other enacted its use. Comparable sequences have been observed in co-speech gestures, where a held depiction maintains reference to an object or character while the other hand introduces related information (Enfield, 2004; Frederiksen, 2016). In recurrent expressions of *contrast*, by comparison, an initial instance of representing was followed by representing with locating, as a spatial relation between the hands was introduced while the comparison unfolded. Although infrequent, these instances suggest that a gestural depiction already produced can serve as a resource for further expression, providing an object or spatial reference to which subsequent gestures are related.

Our study contributes to a broader understanding of how conceptual content is expressed through manual gestures when speakers cannot draw on an established sign language. One possible account draws on selection and schematization, through which features such as an object’s outline or characteristic use are adapted for manual depiction (Taub, 2001; van Nispen et al., 2017). For combinations linking object form with characteristic use, an initial depiction may establish the referent for the subsequent action, consistent with accounts that relate preferences for the order of gestures to semantic dependence (Holtz et al., 2025). A comparable relation can be maintained across the two hands, where a held depiction provides a reference for an action or spatial relation expressed by the other hand (Frederiksen, 2016). In the recurrent pairings of representing with locating and acting with embodying, the relative positions or movements of the hands can additionally depict how an entity is situated or acted upon. For concepts such as *contrast*, a spatial arrangement can also provide a way of depicting comparison, with different positions assigned to the elements being compared. Such correspondences are consistent with accounts of diagrammatic iconicity, in which relations among forms resemble relations among their referents (Taub, 2001; Hodge and Ferrara, 2022). Our findings suggest that familiar relations among entities, actions, and locations may provide a basis for coordinating partial depictions within the expression of an individual concept.

The present study extends the analysis of patterned iconicity (Padden et al., 2013, 2015) by establishing regularities in the relations among meaningful units within gestural expressions. Building on earlier observations of combinatorial patterning, we distinguish the occurrence of combinations from the ways in which their constituent units are arranged. Our findings show that this distinction matters even for closely related categories, as actions with objects and manipulable objects both elicited simultaneous combinations relatively often, whereas sequential combinations were much more characteristic of manipulable objects. Extending these comparisons to abstract meanings revealed a further contrast, with sequential combinations relatively frequent for attributes, social concepts, and cognition, while emotions rarely elicited combinations of manually articulated units. Analyses of position and direction further distinguish frequent co-occurrence from preferred order within recurrent pairs, while the analysis of simultaneous combinations identifies which strategies are repeatedly paired across the hands. The analytical framework developed here connects the iconic strategy assigned to each meaningful unit with its position in a sequence or its pairing across the hands, providing a common basis for comparing the organization of gestures across conceptual categories. In addition, the expanded coding framework incorporates symbolic mapping and schema mapping, providing explicit criteria for examining conventional and schematic contributions alongside other iconic strategies. Applying this framework across multiple abstract categories documents variation within the abstract domain and shows that the organized use of partial depictions extends to meanings without stable visible referents.

## 6 Limitations and future directions

To encourage spontaneous responses, our elicitation task allowed 4 s for participants to read each word and produce a silent gesture response. However, this interval may have limited opportunities for planning, elaboration, or revision, particularly for longer sequences and mixed combinations. The variation in pass responses across conceptual categories also shows that the analyses of combinations concern productions that participants were able to express manually, potentially giving greater weight to concepts that were easier to depict. Therefore, separating the time for reading from the time for production, and varying the latter, would clarify how the time available contributes to pass responses and to the frequency and length of combinations. Our focus on manually articulated units provided a consistent basis for comparing the selection and combination of iconic strategies across conceptual categories. Facial expressions, gaze, mouthing, and movements of the head and torso, however, can convey distinct information alongside the hands and contribute to simultaneous expressions in both silent gesture and sign language (Slonimska et al., 2022). In our data, non-manual-only responses were particularly frequent for emotions and actions without objects, indicating that a focus on the hands may underrepresent these meanings and give greater prominence to resources such as depictions of object form and manipulation. So, extending the analysis to these articulators and their timing would provide a fuller investigation of how meanings are combined and organized in the manual-visual modality.

The present findings characterize a young, university-based, predominantly female, right-handed Mandarin-speaking sample. Broader sampling would clarify the generality of the observed patterns, particularly those involving culturally conventional symbolic mappings. Because the study did not include a signer comparison group, references to sign-language research in the Discussion concern theoretical parallels rather than direct group differences. It is also important to note that some distinctions in the coding scheme were developed after examining the productions, making the associated analyses exploratory (Nosek et al., 2018). Applying the finalized codebook to independently collected gestures would test whether the patterns recur, while participants’ explanations of their gestures could help evaluate our interpretations of their choices. We also see particular value in examining how the combinations observed in gesture production are understood, given that our participants produced gestures for later viewing. Independent viewers could identify the intended concepts without seeing the target words, allowing interpretation to be compared across individual units, complete combinations, and alternative orders (van Nispen et al., 2017). Whereas participants here produced gestures for later viewing, interaction may allow speakers to negotiate meaning and revise their depictions in response to feedback (Ćwiek et al., 2026). Future studies could examine how such negotiation and repair influence the use, ordering, and combination of iconic strategies.

## 7 Conclusion

The present study contributes to an understanding of how hearing non-signers organize iconic representations within the manual expression of individual concepts. Previous research has revealed systematic associations between conceptual categories and iconic strategies (e.g., Hwang et al., 2017; Mamus et al., 2025; Ortega and Özyürek, 2020a), as well as combinations of gestures used to depict individual concepts (Ortega and Özyürek, 2020b; van Nispen et al., 2017). However, identifying which strategies occur together provides only part of an account of their organization. Here, we provide evidence that closely related conceptual categories can draw on similar iconic resources while differing in how those resources are combined, and that recurrent combinations display systematic preferences in their ordering and pairing across the hands. Analyses of preferred order and recurrent pairings further showed that these expressions have regularities in the relations among their constituent units. An important implication is that familiar relations among entities, actions, and locations may provide a basis for coordinating partial depictions, allowing an object or spatial reference established by one gesture to participate in the representation contributed by another. Overall, these findings extend earlier accounts of patterned iconicity (Padden et al., 2013, 2015) by showing how conceptual distinctions are expressed through the organization of combinations, providing an empirical basis for relating the use of iconic strategies to the temporal and spatial relations through which they jointly represent a concept in the manual-visual modality.

## Data Availability

The anonymized analysis-ready data supporting the findings of this study are provided as Supplementary material in Anonymized_Analysis_Data.xlsx. Supplementary result tables are provided in Supplementary_Materials.xlsx, coding criteria and decision rules are provided in the Supplementary_Codebook.pdf, and the analysis script is provided in Reproducible_Analysis.R. Raw video recordings are not publicly available because they contain identifiable participant information. Additional information about the analyses is available from the corresponding author upon reasonable request.

## Footnotes

1 Strongly context-dependent items were those whose intended interpretation could not be reliably identified from the isolated lexical form because an argument, referent, comparison standard, or situational context was required (Barsalou, 1982). Operationally, in the present study, an item was excluded if presenting it without a sentence or situational context did not yield a single self-contained interpretation, and the plausible interpretations would support substantially different gesture productions. For example, dāngdì (‘local’) requires a relevant location, whereas héshì (‘suitable’) requires a purpose or comparison standard.

## References

[ref1] Alcalá-López D. Mei N. Margolles P. Soto D. (2024). Brain-wide representation of social knowledge. Soc. Cogn. Affect. Neurosci. 19:nsae032. doi: 10.1093/scan/nsae032, 38804694 PMC11173195

[ref2] Altarriba J. Bauer L. M. (2004). The distinctiveness of emotion concepts: a comparison between emotion, abstract, and concrete words. Am. J. Psychol. 117, 389–410. doi: 10.2307/4149007 15457808

[ref3] Altarriba J. Bauer L. M. Benvenuto C. (1999). Concreteness, context availability, and imageability ratings and word associations for abstract, concrete, and emotion words. Behav. Res. Methods Instrum. Comput. 31, 578–602. doi: 10.3758/BF03200738, 10633977

[ref4] Aronoff M. Meir I. Sandler W. (2005). The paradox of sign language morphology. Language 81, 301–344. doi: 10.1353/lan.2005.0043, 22223926 PMC3250214

[ref5] Banks B. Connell L. (2023). Multi-dimensional sensorimotor grounding of concrete and abstract categories. Philos. Trans. R. Soc. B Biol. Sci. 378:Article 20210366. doi: 10.1098/rstb.2021.0366, 36571121 PMC9791484

[ref6] Barsalou L. W. (1982). Context-independent and context-dependent information in concepts. Mem. Cogn. 10, 82–93. doi: 10.3758/BF03197629, 7087773

[ref7] Barsalou L. W. (2005). “Situated conceptualization,” in Handbook of Categorization in Cognitive Science, eds. Cohen H. Lefebvre C. (Amsterdam, The Netherlands: Elsevier), 619–650.

[ref8] Bates D. Mächler M. Bolker B. Walker S. (2015). Fitting linear mixed-effects models using lme4. J. Stat. Softw. 67, 1–48. doi: 10.18637/jss.v067.i01

[ref9] Bohn M. Kachel G. Tomasello M. (2019). Young children spontaneously recreate core properties of language in a new modality. Proc. Natl. Acad. Sci. 116, 26072–26077. doi: 10.1073/pnas.1904871116, 31792169 PMC6926062

[ref10] Borghi A. M. Binkofski F. Castelfranchi C. Cimatti F. Scorolli C. Tummolini L. (2017). The challenge of abstract concepts. Psychol. Bull. 143, 263–292. doi: 10.1037/bul0000089, 28095000

[ref11] Borghi A. M. Capirci O. Gianfreda G. Volterra V. (2014). The body and the fading away of abstract concepts and words: a sign language analysis. Front. Psychol. 5:811. doi: 10.3389/fpsyg.2014.00811, 25120515 PMC4114187

[ref12] Börstell C. Lepic R. (2025). Non-signers favor two-handed gestures when expressing inherently plural meanings. Linguist. Vanguard 11, 545–557. doi: 10.1515/lingvan-2024-0181

[ref13] Bradley C. Wilbur R. (2023). Visual form and event semantics predict transitivity in silent gestures: evidence for compositionality. Cogn. Sci. 47:e13331. doi: 10.1111/cogs.13331, 37635624

[ref14] Brentari D. (2002). “Modality differences in sign language phonology and morphophonemics,” in Modality and Structure in Signed and Spoken Languages, eds. Meier R. P. Cormier K. Quinto-Pozos D. (Cambridge, UK: Cambridge University Press), 35–64.

[ref15] Brentari D. (2019). Sign Language Phonology. New York, NY, USA: Cambridge University Press.

[ref16] Brentari D. Di Renzo A. Keane J. Volterra V. (2015). Cognitive, cultural, and linguistic sources of a handshape distinction expressing agentivity. Top. Cogn. Sci. 7, 95–123. doi: 10.1111/tops.12123, 25529989

[ref17] Brentari D. Goldin-Meadow S. Horton L. Senghas A. Coppola M. (2024). The organization of verb meaning in Lengua de Señas Nicaragüense (LSN): sequential or simultaneous structures? Glossa J. Gen. Linguist. 9, 1–37. doi: 10.16995/glossa.10342 PMC12356425 40822377

[ref18] Brentari D. Horton L. Goldin-Meadow S. (2020). Crosslinguistic similarity and variation in the simultaneous morphology of sign languages. Linguist. Rev. 37, 571–608. doi: 10.1515/tlr-2020-2055

[ref19] Brooks M. E. Kristensen K. van Benthem K. J. Magnusson A. Berg C. W. Nielsen A. . (2017). GlmmTMB balances speed and flexibility among packages for zero-inflated generalized linear mixed modeling. R J. 9, 378–400. doi: 10.32614/RJ-2017-066

[ref20] Cai Q. Brysbaert M. (2010). SUBTLEX-CH: Chinese word and character frequencies based on film subtitles. PLoS One 5:e10729. doi: 10.1371/journal.pone.0010729, 20532192 PMC2880003

[ref21] Christensen P. Fusaroli R. Tylén K. (2016). Environmental constraints shaping constituent order in emerging communication systems: structural iconicity, interactive alignment and conventionalization. Cognition 146, 67–80. doi: 10.1016/j.cognition.2015.09.004, 26402649

[ref22] Cienki A. (2013). Image schemas and mimetic schemas in cognitive linguistics and gesture studies. Rev. Cogn. Linguist. 11, 417–432. doi: 10.1075/rcl.11.2.13cie

[ref23] Conca F. Borsa V. M. Cappa S. F. Catricalà E. (2021). The multidimensionality of abstract concepts: a systematic review. Neurosci. Biobehav. Rev. 127, 474–491. doi: 10.1016/j.neubiorev.2021.05.004, 33979574

[ref24] Cormier K. Fenlon J. Schembri A. (2015). Indicating verbs in British sign language favour motivated use of space. Open Linguist. 1, 684–707. doi: 10.1515/opli-2015-0025, 31755547

[ref25] Cormier K. Smith S. Sevcikova Z. (2013). Predicate structures, gesture, and simultaneity in the representation of action in British sign language: evidence from deaf children and adults. J. Deaf Stud. Deaf Educ. 18, 370–390. doi: 10.1093/deafed/ent020, 23670881 PMC3943391

[ref26] Crutch S. J. Warrington E. K. (2005). Abstract and concrete concepts have structurally different representational frameworks. Brain 128, 615–627. doi: 10.1093/brain/awh349 15548554

[ref27] Ćwiek A. Fuchs S. Pouw W. Kadavá Š. (2026). Self-reported expressibility predicts communicative success: open dataset, validation, and simulation. Behav. Res. Methods 58:196. doi: 10.3758/s13428-026-03071-4, 42283796 PMC13263253

[ref28] Dingemanse M. Blasi D. E. Lupyan G. Christiansen M. H. Monaghan P. (2015). Arbitrariness, iconicity, and systematicity in language. Trends Cogn. Sci. 19, 603–615. doi: 10.1016/j.tics.2015.07.013, 26412098

[ref29] Dingemanse M. Perlman M. Perniss P. (2020). Construals of iconicity: experimental approaches to form–meaning resemblances in language. Lang. Cogn. 12, 1–14. doi: 10.1017/langcog.2019.48

[ref30] Diveica V. Muraki E. J. Binney R. J. Pexman P. M. (2024). Mapping semantic space: exploring the higher-order structure of word meaning. Cognition 248:105794. doi: 10.1016/j.cognition.2024.105794, 38653181

[ref31] Dudis P. G. (2004). Body partitioning and real-space blends. Cogn. Linguist. 15, 223–238. doi: 10.1515/cogl.2004.009

[ref32] Ekman P. Friesen W. V. (1969). The repertoire of nonverbal behavior: categories, origins, usage, and coding. Semiotica 1, 49–98. doi: 10.1515/semi.1969.1.1.49

[ref33] Emmorey K. (2002). Language, Cognition, and the Brain: Insights from sign Language Research. Mahwah, NJ, USA: Lawrence Erlbaum Associates.

[ref34] Emmorey K. (2014). Iconicity as structure mapping. Philos. Trans. R. Soc. B Biol. Sci. 369:20130301. doi: 10.1098/rstb.2013.0301, 25092669 PMC4123680

[ref35] Enfield N. J. (2004). On linear segmentation and combinatorics in co-speech gesture: a symmetry-dominance construction in Lao fish trap descriptions. Semiotica 149, 57–123. doi: 10.1515/semi.2004.038

[ref36] Engberg-Pedersen E. Brentari D. K. Davis J. (2025). Formal and functional factors in classifier choice: evidence from American sign language and Danish sign language. Open Linguist. 11:Article 20250043. doi: 10.1515/opli-2025-0043

[ref37] Fay N. Ellison M. Garrod S. (2014). Iconicity: from sign to system in human communication and language. Pragmat. Cogn. 22, 244–263. doi: 10.1075/pc.22.2.05fay

[ref38] Fillmore C. J. (1982). “Frame semantics,” in Linguistics in the Morning calm, (Seoul, South Korea: Hanshin Publishing), 111–137.

[ref39] Fischer S. D. Janis W. (1990). “Verb sandwiches in American sign language,” in Theoretical Issues in sign Language Research: Vol. 1. Linguistics, eds. Fischer S. D. Siple P. (Chicago, IL, USA: University of Chicago Press), 279–293.

[ref40] Flaherty M. Schouwstra M. (2025). Validating silent gesture lab studies in a naturally emerging sign language: how order is used to describe intensional versus extensional events in Nicaraguan sign language. Top. Cogn. Sci. 17, 609–624. doi: 10.1111/tops.12751, 39190828

[ref41] Frederiksen A. T. (2016). Hold + stroke gesture sequences as cohesion devices: examples from Danish narratives. San Diego Linguist. Pap. 6, 2–13.

[ref42] Frederiksen A. T. (2017). Separating viewpoint from mode of representation in iconic co-speech gestures: insights from Danish narratives. Lang. Cogn. 9, 677–708. doi: 10.1017/langcog.2016.35

[ref43] Gawne L. Cooperrider K. (2024). Emblems: meaning at the interface of language and gesture. Glossa J. Gen. Linguist. 9, 1–39. doi: 10.16995/glossa.9705

[ref44] Gentner D. Markman A. B. (1997). Structure mapping in analogy and similarity. Am. Psychol. 52, 45–56. doi: 10.1037/0003-066X.52.1.45

[ref45] Goldin-Meadow S. McNeill D. Singleton J. (1996). Silence is liberating: removing the handcuffs on grammatical expression in the manual modality. Psychol. Rev. 103, 34–55. doi: 10.1037/0033-295X.103.1.34, 8650298

[ref46] Goldin-Meadow S. So W. C. Özyürek A. Mylander C. (2008). The natural order of events: how speakers of different languages represent events nonverbally. Proc. Natl. Acad. Sci. 105, 9163–9168. doi: 10.1073/pnas.0710060105, 18599445 PMC2453738

[ref47] Gulamani S. Marshall C. Morgan G. (2022). The challenges of viewpoint-taking when learning a sign language: data from the ‘frog story’ in British sign language. Second. Lang. Res. 38, 55–87. doi: 10.1177/0267658320906855

[ref48] Hall M. L. Mayberry R. I. Ferreira V. S. (2013). Cognitive constraints on constituent order: evidence from elicited pantomime. Cognition 129, 1–17. doi: 10.1016/j.cognition.2013.05.004, 23792806 PMC4224279

[ref49] Harpaintner M. Trumpp N. M. Kiefer M. (2018). The semantic content of abstract concepts: a property listing study of 296 abstract words. Front. Psychol. 9:Article 1748. doi: 10.3389/fpsyg.2018.01748, 30283389 PMC6156367

[ref50] Hassemer J. Winter B. (2018). Decoding gestural iconicity. Cogn. Sci. 42, 3034–3049. doi: 10.1111/cogs.12680, 30471078

[ref51] Heesen R. Kim Y. Kret M. E. Clay Z. (2024). Perceptual integration of bodily and facial emotion cues in chimpanzees and humans. PNAS Nexus 3:Article pgae012. doi: 10.1093/pnasnexus/pgae012, 38344008 PMC10855020

[ref52] Hodge G. Ferrara L. (2022). Iconicity as multimodal, polysemiotic, and plurifunctional. Front. Psychol. 13:808896. doi: 10.3389/fpsyg.2022.808896, 35769755 PMC9234520

[ref53] Holtz A. Kirby S. Culbertson J. (2025). With or without a system: how category-specific and system-wide cognitive biases shape word order. Cogn. Sci. 49:e70139. doi: 10.1111/cogs.70139, 41305994 PMC12658409

[ref54] Hostetter A. B. Alibali M. W. (2008). Visible embodiment: gestures as simulated action. Psychon. Bull. Rev. 15, 495–514. doi: 10.3758/PBR.15.3.495, 18567247

[ref55] Hostetter A. B. Alibali M. W. (2019). Gesture as simulated action: revisiting the framework. Psychon. Bull. Rev. 26, 721–752. doi: 10.3758/s13423-018-1548-0, 30511231

[ref56] Hou L. (2018). Iconic patterns in San Juan Quiahije Chatino sign language. Sign Lang. Stud. 18, 570–611. doi: 10.1353/sls.2018.0017

[ref57] Hwang S.-O. Tomita N. Morgan H. Ergin R. İlkbaşaran D. Seegers S. . (2017). Of the body and the hands: patterned iconicity for semantic categories. Lang. Cogn. 9, 573–602. doi: 10.1017/langcog.2016.28

[ref58] Jo C. (2026). The dynamic interplay of metaphor and iconicity in Korean sign language. Linguist. Vanguard. doi: 10.1515/lingvan-2025-0122

[ref59] Johnson M. (1987). The body in the mind: The Bodily basis of Meaning, Imagination, and Reason. Chicago, IL, USA: University of Chicago Press.

[ref60] Kellenbach M. L. Brett M. Patterson K. (2003). Actions speak louder than functions: the importance of manipulability and action in tool representation. J. Cogn. Neurosci. 15, 30–46. doi: 10.1162/089892903321107800, 12590841

[ref61] Kendon A. (2004). Gesture: Visible Action as Utterance. Cambridge, UK: Cambridge University Press.

[ref62] Keränen J. (2021). Iconic strategies in lexical sensory signs in Finnish sign language. Cogn. Semiot. 14, 163–187. doi: 10.1515/cogsem-2021-2042

[ref63] Kimmelman V. Klezovich A. Moroz G. (2018). IPSL: A database of iconicity patterns in sign languages. Creation and use Calzolari N. Choukri K. Cieri C. Declerck T. Goggi S. . Proceedings of the Eleventh International Conference on Language Resources and Evaluation (LREC 2018). Miyazaki, Japan: European Language Resources Association. Available online at: https://aclanthology.org/L18-1667/ (Accessed October 8, 2026).

[ref64] Lakoff G. Johnson M. (1980). Metaphors we live by. Chicago, IL, USA: University of Chicago Press.

[ref65] Langacker R. W. (1993). Reference-point constructions. Cogn. Linguist. 4, 1–38. doi: 10.1515/cogl.1993.4.1.1

[ref66] Langacker R. W. (2008). Cognitive Grammar: A basic Introduction. New York, NY, USA: Oxford University Press. doi: 10.1093/acprof:oso/9780195331967.001.0001.

[ref67] Lenth R. V. Piaskowski J. (2025). Emmeans: Estimated marginal means, aka least-Squares means (R Package Version 2.0.0) [R Package]. doi: 10.32614/CRAN.package.emmeans.

[ref68] Lepic R. Börstell C. Belsitzman G. Sandler W. (2016). Taking meaning in hand: iconic motivations in two-handed signs. Sign Lang. Linguist. 19, 37–81. doi: 10.1075/sll.19.1.02lep

[ref69] Loos C. German A. Meier R. P. (2022). Simultaneous structures in sign languages: Acquisition and emergence. Front. Psychol. 13:992589. doi: 10.3389/fpsyg.2022.992589, 36619119 PMC9815181

[ref70] Mamus E. Speed L. J. Ortega G. Majid A. Özyürek A. (2025). Gestural and verbal evidence of conceptual representation differences in blind and sighted individuals. Cogn. Sci. 49:e70125. doi: 10.1111/cogs.70125, 41082278 PMC12517398

[ref71] Masson-Carro I. Goudbeek M. Krahmer E. (2016). Can you handle this? The impact of object affordances on how co-speech gestures are produced. Lang. Cogn. Neurosci. 31, 430–440. doi: 10.1080/23273798.2015.1108448, 27226970 PMC4867791

[ref72] Masson-Carro I. Goudbeek M. Krahmer E. (2017). How what we see and what we know influence iconic gesture production. J. Nonverbal Behav. 41, 367–394. doi: 10.1007/s10919-017-0261-4, 29104335 PMC5649601

[ref73] Meier R. P. (2012). “Language and modality,” in Sign Language: An International Handbook, eds. Pfau R. Steinbach M. Woll B. (Berlin, Germany: De Gruyter Mouton), 574–601. doi: 10.1515/9783110261325.574

[ref74] Meir I. (2010). Iconicity and metaphor: constraints on metaphorical extension of iconic forms. Language 86, 865–896. doi: 10.1353/lan.2010.0044

[ref75] Mittelberg I. (2017). Embodied frames and scenes: body-based metonymy and pragmatic inferencing in gesture. Gesture 16, 203–236. doi: 10.1075/gest.16.2.03mit

[ref76] Mittelberg I. (2018). Gestures as image schemas and force gestalts: a dynamic systems approach augmented with motion-capture data analyses. Cogn. Semiot. 11:Article 20180002. doi: 10.1515/cogsem-2018-0002

[ref77] Mittelberg I. (2019). Visuo-kinetic signs are inherently metonymic: how embodied metonymy motivates forms, functions, and schematic patterns in gesture. Front. Psychol. 10:254. doi: 10.3389/fpsyg.2019.00254, 30873059 PMC6400971

[ref78] Mittelberg I. Waugh L. R. (2014). “Gestures and metonymy,” in Body – Language – Communication: An International Handbook on Multimodality in human Interaction, eds. Müller C. Cienki A. Fricke E. Ladewig S. H. McNeill D. Bressem J., vol. 2 (Berlin, Germany: De Gruyter Mouton), 1747–1766.

[ref79] Morgan G. (2014). On language acquisition in speech and sign: development of combinatorial structure in both modalities. Front. Psychol. 5:Article 1217. doi: 10.3389/fpsyg.2014.01217, 25426085 PMC4227467

[ref80] Müller C. (2014). “Gestural modes of representation as techniques of depiction,” in Body – Language – Communication: An International Handbook on Multimodality in human Interaction, eds. Müller C. Cienki A. Fricke E. Ladewig S. H. McNeill D. Bressem J., vol. 2 (Berlin, Germany: De Gruyter Mouton), 1687–1701.

[ref81] Nakamura C. Zeng-Treitler Q. (2012). A taxonomy of representation strategies in iconic communication. Int. J. Human-Comp. Stud. 70, 535–551. doi: 10.1016/j.ijhcs.2012.02.009, 22754274 PMC3383821

[ref82] Nosek B. A. Ebersole C. R. DeHaven A. C. Mellor D. T. (2018). The preregistration revolution. Proc. Natl. Acad. Sci. 115, 2600–2606. doi: 10.1073/pnas.1708274114, 29531091 PMC5856500

[ref83] Nyst V. Morgado M. Hadjah T. M. Nyarko M. Martins M. van der Mark L. . (2022). Object and handling handshapes in 11 sign languages: towards a typology of the iconic use of the hands. Linguist. Typol. 26, 573–604. doi: 10.1515/lingty-2021-0026

[ref84] Ortega G. Özyürek A. (2020a). Systematic mappings between semantic categories and types of iconic representations in the manual modality: a normed database of silent gesture. Behav. Res. Methods 52, 51–67. doi: 10.3758/s13428-019-01204-6, 30788798 PMC7005091

[ref85] Ortega G. Özyürek A. (2020b). Types of iconicity and combinatorial strategies distinguish semantic categories in silent gesture across cultures. Lang. Cogn. 12, 84–113. doi: 10.1017/langcog.2019.28

[ref86] Ortega G. Schiefner A. Lazarus N. Perniss P. (2025). A lexical database of British sign language (BSL) and German sign language (DGS): iconicity ratings, iconic strategies, and concreteness norms. Behav. Res. Methods 57:139. doi: 10.3758/s13428-025-02660-z, 40205134 PMC11982127

[ref87] Ortega G. Schiefner A. Özyürek A. (2019). Hearing non-signers use their gestures to predict iconic form-meaning mappings at first exposure to signs. Cognition 191:103996. doi: 10.1016/j.cognition.2019.06.008, 31238248

[ref88] Özçalışkan Ş. Lucero C. Goldin-Meadow S. (2016). Does language shape silent gesture? Cognition 148, 10–18. doi: 10.1016/j.cognition.2015.12.001, 26707427 PMC4724526

[ref89] Padden C. Hwang S.-O. Lepic R. Seegers S. (2015). Tools for language: patterned iconicity in sign language nouns and verbs. Top. Cogn. Sci. 7, 81–94. doi: 10.1111/tops.12121, 25408329

[ref90] Padden C. A. Meir I. Hwang S.-O. Lepic R. Seegers S. Sampson T. (2013). Patterned iconicity in sign language lexicons. Gesture 13, 287–308. doi: 10.1075/gest.13.3.03pad

[ref91] Peirce J. W. Gray J. R. Simpson S. MacAskill M. R. Höchenberger R. Sogo H. . (2019). PsychoPy2: experiments in behavior made easy. Behav. Res. Methods 51, 195–203. doi: 10.3758/s13428-018-01193-y, 30734206 PMC6420413

[ref92] Perniss P. (2007). Space and iconicity in German Sign Language (DGS) [Doctoral dissertation, Radboud University Nijmegen].

[ref93] Perniss P. Özyürek A. Morgan G. (2015). The influence of the visual modality on language structure and conventionalization: insights from sign language and gesture. Top. Cogn. Sci. 7, 2–11. doi: 10.1111/tops.12127, 25565249

[ref94] Perniss P. Thompson R. Vigliocco G. (2010). Iconicity as a general property of language: evidence from spoken and signed languages. Front. Psychol. 1:227. doi: 10.3389/fpsyg.2010.00227, 21833282 PMC3153832

[ref95] Perniss P. Vigliocco G. (2014). The bridge of iconicity: from a world of experience to the experience of language. Philos. Trans. R. Soc. Lond. Ser. B Biol. Sci. 369:20130300. doi: 10.1098/rstb.2013.0300, 25092668 PMC4123679

[ref96] Placiński M. Żywiczyński P. Matzinger T. Sibierska M. Boruta-Żywiczyńska M. Szala A. . (2023). Evolution of pantomime in dyadic interaction. A motion capture study. J. Lang. Evol. 8, 134–148. doi: 10.1093/jole/lzad010

[ref97] Poggi I. (2008). Iconicity in different types of gestures. Gesture 8, 45–61. doi: 10.1075/gest.8.1.05pog

[ref98] Pouw W. Dingemanse M. Motamedi Y. Özyürek A. (2021). A systematic investigation of gesture kinematics in evolving manual languages in the lab. Cogn. Sci. 45:e13014. doi: 10.1111/cogs.13014, 34288069 PMC8365719

[ref99] R Core Team. (2025). R: A Language and Environment for Statistical Computing. Vienna, Austria: R Foundation for Statistical Computing. Available online at: https://www.R-project.org/

[ref100] Recchia G. Jones M. N. (2012). The semantic richness of abstract concepts. Front. Hum. Neurosci. 6:315. doi: 10.3389/fnhum.2012.00315, 23205008 PMC3506984

[ref101] Schiefner A. (2025). Patterns of iconicity across sign languages and gestures [doctoral dissertation, Universität zu Köln]. Kölner UniversitätsPublikationsServer. Available online at: https://kups.ub.uni-koeln.de/78615/ (Accessed October 8, 2026).

[ref102] Schouwstra M. de Swart H. (2014). The semantic origins of word order. Cognition 131, 431–436. doi: 10.1016/j.cognition.2014.03.004, 24704967

[ref103] Senghas A. Kita S. Özyürek A. (2004). Children creating core properties of language: evidence from an emerging sign language in Nicaragua. Science 305, 1779–1782. doi: 10.1126/science.1100199, 15375269

[ref104] Slonimska A. Özyürek A. Capirci O. (2020). The role of iconicity and simultaneity for efficient communication: the case of Italian sign language (LIS). Cognition 200:104246. doi: 10.1016/j.cognition.2020.104246, 32197151

[ref105] Slonimska A. Özyürek A. Capirci O. (2022). Simultaneity as an emergent property of efficient communication in language: a comparison of silent gesture and sign language. Cogn. Sci. 46:e13133. doi: 10.1111/cogs.13133, 35613353 PMC9287048

[ref106] Supalla T. (1982). Structure and acquisition of verbs of motion and location in American Sign Language [Doctoral dissertation, University of California, San Diego].

[ref107] Supalla T. (1990). “Serial verbs of motion in ASL,” in Theoretical Issues in Sign Language Research: Vol. 1. Linguistics, eds. Fischer S. D. Siple P. (Chicago, IL, USA: University of Chicago Press), 127–152.

[ref108] Talmy L. (2000). Toward a Cognitive Semantics, vol. 1–2. Cambridge, MA, USA: MIT Press.

[ref109] Taub S. F. (1997). Language in the body: Iconicity and Metaphor in American Sign Language [Doctoral Dissertation, University of California, Berkeley].

[ref110] Taub S. F. (2001). Language from the body: Iconicity and Metaphor in American Sign Language. Cambridge, UK: Cambridge University Press.

[ref111] Ünal E. Kırbaşoğlu K. Karadöller D. Z. Sümer B. Özyürek A. (2025). Gesture reduces mapping difficulties in the development of spatial language depending on the complexity of spatial relations. Cogn. Sci. 49:e70046. doi: 10.1111/cogs.70046, 39992958 PMC11849910

[ref112] van der Kooij E. Zwitserlood I. Crasborn O. (2023). Strategies for new word formation in NGT: a case for simultaneous morphology. Sign Lang. Linguist. 26, 176–217. doi: 10.1075/sll.20001.van

[ref113] van Nispen K. van de Sandt-Koenderman W. M. E. Krahmer E. (2017). Production and comprehension of pantomimes used to depict objects. Front. Psychol. 8:1095. doi: 10.3389/fpsyg.2017.01095, 28744232 PMC5504161

[ref114] Vermeerbergen M. Leeson L. Crasborn O. (2007). Simultaneity in Signed Languages. Amsterdam, The Netherlands: John Benjamins.

[ref115] Villani C. Lugli L. Liuzza M. T. Borghi A. M. (2019). Varieties of abstract concepts and their multiple dimensions. Lang. Cogn. 11, 403–430. doi: 10.1017/langcog.2019.23

[ref116] Villani C. Lugli L. Liuzza M. T. Nicoletti R. Borghi A. M. (2021). Sensorimotor and interoceptive dimensions in concrete and abstract concepts. J. Mem. Lang. 116:104173. doi: 10.1016/j.jml.2020.104173, 32952286 PMC7492812

[ref117] Wittenburg P. Brugman H. Russel A. Klassmann A. Sloetjes H. (2006). ELAN: A professional framework for multimodality research Calzolari N. Choukri K. Gangemi A. Maegaard B. Mariani J. Odijk J. . Proceedings of the Fifth International Conference on Language Resources and Evaluation (LREC’06) European Language Resources Association Available online at: https://aclanthology.org/L06-1082/ (Accessed October 8, 2026).

[ref118] Zacks J. M. Tversky B. (2001). Event structure in perception and conception. Psychol. Bull. 127, 3–21. doi: 10.1037/0033-2909.127.1.3, 11271755

[ref119] Zeileis A. Köll S. Graham N. (2020). Various versatile variances: an object-oriented implementation of clustered covariances in R. J. Stat. Softw. 95, 1–36. doi: 10.18637/jss.v095.i01

[ref120] Zlatev J. Żywiczyński P. Wacewicz S. (2020). Pantomime as the original human-specific communicative system. J. Lang. Evol. 5, 156–174. doi: 10.1093/jole/lzaa006

