# Supplementary material for: Combinatorial structure of iconic strategies in the manual-visual modality: evidence from silent gestures by hearing non-signers
Source: Front Psychol. 2026 Oct 16;17:1917740. doi: 10.3389/fpsyg.2026.1917740 (PMC13719605; doi:10.3389/fpsyg.2026.1917740)
Supplement: Supplementary file 1 [file Data_Sheet_1.PDF]

Supplementary Codebook for Coding Iconic Strategies and Combinatorial Structures in Silent Gestures

1. Overview and Development of the Coding Scheme

This codebook documents the coding decisions needed to understand and reproduce the coding reported in the manuscript. It focuses on response screening, segmentation into meaningful gesture units, assignment of iconic strategies and iconicity types, classification of combinatorial structure, and the structural variables derived from sequential and simultaneous responses. Statistical model specifications and supplementary result tables are provided separately and are not repeated here.

Coding proceeded in three stages. First, each trial was screened for whether it contained analyzable manual gesture. Second, each manual response was segmented into meaningful gesture units and each unit was assigned a fine-grained iconic strategy and a broader iconicity type. Third, each response was classified as non-combinatorial, sequential-only, simultaneous-only, or mixed according to the number and organization of its meaningful units.

The initial inventory was adapted from previous classifications of iconic representation in gesture and sign language, especially work distinguishing action-based, form-based, and spatial or relational strategies (Müller, 2014; Padden et al., 2015; Hwang et al., 2017; Ortega & Özyürek, 2020a, 2020b; van Nispen et al., 2017). This initial inventory contained nine strategies. During coding, two recurrent sets of units were not adequately captured by that inventory. Symbolic mapping and schema mapping were therefore added after inspection of the productions. Their interpretation was informed by research on conventional gestures and emblems and on image-schematic representation (Kendon, 2004; Poggi, 2008; Cienki, 2013; Mittelberg, 2018). These two categories are data-informed extensions and should be interpreted as exploratory additions to the coding framework. Full references are provided in the manuscript.

Where to look	Rule covered
Section 2	Pass, non-manual-only, manual responses, meaningful unit boundaries, repetitions, abandoned movements, and bimanual segmentation
Section 3	Difference between iconic strategy and iconicity type, plus which categories were pre-defined and which were added after inspection
Section 4	Operational definitions, examples, and the main boundary decisions for all 11 iconic strategies
Section 5	Non-combinatorial, sequential-only, simultaneous-only, and mixed response categories
Section 6	Focal and anchoring hand roles, sequential position, co-occurrence, transitions, and treatment of mixed responses
Section 7	Use of other and exclusions from structural analyses
Section 8	Worked coding examples and interpretation limits

2. Response Screening and Meaningful Gesture Units

Each trial corresponded to one participant response to one target concept. Screening was performed before strategy coding so that pass responses and responses without a manual component were distinguished from analyzable manual responses.

Response status	Operational rule	Examples and exclusions
Pass	No gesture or no completed communicative movement was produced for the target concept.	Includes explicit pass, remaining still, or an abandoned preparation with no identifiable semantic contribution. A brief manual gesture with identifiable content was not coded as pass.

Response status	Operational rule	Examples and exclusions
Non-manual-only	Meaning was conveyed without an observable manual gesture unit.	Includes facial expression, head movement, gaze, torso movement, or whole-body posture without a manual component. If the hands contributed identifiable content, the response was coded as manual.
Manual response	At least one manual component contributed identifiable semantic or representational content.	Manual responses entered the main coding of meaningful units and combination structure. Non-manual behavior accompanying a manual gesture was treated as contextual information and was not coded as an independent unit.

2.1 Meaningful unit definition and boundaries

A meaningful gesture unit was the smallest manually articulated component that contributed identifiable semantic content to a response. Unit boundaries were based on representational content rather than on the response as a whole.

- A new unit was identified when one completed semantic contribution was followed by another distinguishable semantic contribution.
- Transitional movements, preparation movements, returns to rest, self-adjustments, and hesitations were not coded as separate units.
- A repetition that added no semantic content remained part of the same unit. Repeated or similar movements were separated only when they contributed distinguishable semantic information.
- An abandoned movement was not retained if no identifiable semantic contribution was completed. If a first meaningful unit was completed and a second completed unit contributed different information, both units were retained. The coding did not infer whether the second unit reflected repair or correction.
- Two hands moving together did not automatically constitute two units. If both hands jointly formed one integrated depiction, they were coded as one unit. If the two hands simultaneously encoded distinguishable semantic components, they were coded as two simultaneous units.

3. Iconic Strategies and Iconicity Types

Each valid meaningful unit received two linked labels. Iconic strategy identified the specific representational operation used by the unit. Iconicity type identified the broader form-meaning relation shared by strategies that relied on a comparable representational resource. The type label was therefore a grouping variable, not a second competing strategy label. Every valid iconic strategy mapped to one iconicity type.

Iconicity type	Fine-grained strategies	Central relation	Status in coding scheme
Action-based	Acting; Enacting	Meaning is represented through manual action or bodily reenactment.	Pre-defined from previous gesture and sign-language classifications.
Form-based	Drawing; Embodying; Representing; Personification	Meaning is represented through visible form, entity structure, or mapping between bodily form and referent.	Pre-defined from previous gesture and sign-language classifications.
Relation-based	Pointing; Locating; Path mapping	Meaning is represented through indication, spatial placement, or trajectory.	Pre-defined from previous gesture and sign-language classifications.
Convention-based	Symbolic mapping	A culturally recognizable manual form contributes through a conventional association.	Added after inspection of recurrent productions.
Schema-based	Schema mapping	A schematic spatial or dynamic relation provides the main correspondence with the target meaning.	Added after inspection of recurrent productions.

The distinction between the last three types is especially important. Relation-based strategies encode a locus, placement, or trajectory. Convention-based iconicity depends on a culturally familiar form-meaning association.

Schema-based iconicity requires an identifiable schematic relation such as containment, balance, force, scale, cycle, opposition, or center-periphery. Schema mapping was not used as a residual category for abstract or difficult gestures. When no defined strategy could be assigned reliably, the unit was coded as other as specified in Section 7.

4. Operational Definitions and Boundary Rules

Representative visual examples of all 11 strategies are provided in Figure 1 of the manuscript. The table below gives the operational rule, text examples, and the main distinction used when neighboring categories were plausible.

4.1 Operational definitions of each strategy

Strategy	Code when	Examples	Key boundary
Acting	The hand or hands simulate a specific manual action, object-directed manipulation, or typical use of an object.	Mimicking cutting, writing, stirring, hammering, pressing a button, or holding and using an absent tool.	Use enacting for broader bodily action or state without specific object manipulation. Use representing when the hand functions mainly as the object itself.
Enacting	The hands participate in the reenactment of a broader activity, bodily state, or emotional experience.	Pretending to sleep, shivering, crying, swimming, or adopting a bodily posture associated with prayer.	Use acting when the central information is a specific manual manipulation. Use personification when the gesturer maps their body onto a non-human referent.
Drawing	The hand dynamically traces the outline, contour, boundary, or visible shape of a referent in gesture space.	Tracing a circle for a plate, a rectangle for a laptop, or an arch for a bridge.	Use path mapping when the movement itself represents a route or trajectory rather than an object contour.
Embodying	The handshape or hand configuration instantiates the form or structural arrangement of a referent.	Spreading fingers for spider legs, curving hands for a bowl, or shaping fingers to depict glasses.	Use representing when the hand functions mainly as a token for an entity rather than when its internal form is the primary source of resemblance.
Representing	The hand functions as a proxy or token for an entity, object, person, or participant in a represented scene.	Two fingers standing for a person, one hand moving as a vehicle, or one hand standing for an object while the other acts on it.	Use embodying when hand form itself is central. Use locating when placement in space is the main semantic contribution.
Personification	The gesturer maps their own body or body parts onto the body or body part of a non-human referent.	Hands near the head for animal ears or horns, arms as wings, or an arm as an elephant trunk.	Use enacting for a human action or state. Use representing when a hand is merely a small token for an animal or entity in space.
Pointing	The hand indicates or selects a locus, referent, direction, or contrast point rather than depicting the referent itself.	Pointing to the head, to a location in space, or to two loci to establish contrast.	Use locating when a represented entity is placed at a locus. Use path mapping when the movement traces a route.
Locating	An entity or meaningful component is placed at a specific location in gesture space, establishing a spatial relation or local ground.	Holding one hand as a stable ground while another hand moves above it, or placing two represented entities apart.	Use pointing when the gesture only indicates a place. Use representing when entityhood, rather than placement, is the main contribution.
Path mapping	The hand movement itself represents a trajectory, route, transition, or directional relation between locations.	Moving along a winding route for a river or road, showing travel from one locus to another, or tracing a crossing trajectory.	Use drawing for a visible contour. Use schema mapping only when the movement expresses an abstract schematic relation rather than a concrete or imagined route.
Symbolic mapping	A culturally recognizable manual form contributes through a conventional association that retains an identifiable iconic or metaphorical motivation.	Thumbs-up for positive evaluation, a heart gesture for love, or clasped hands used as a conventional symbol of hope or gratitude.	This label does not treat emblem as a separate communicative class. If the form primarily reenacts an action, use acting or enacting. If it primarily indicates a locus, use pointing.

Strategy	Code when	Examples	Key boundary
Schema mapping	A specific schematic spatial or dynamic relation provides the principal correspondence with a less directly depictable meaning.	Circular motion for recurrence, balanced hands for fairness, enclosing movement for containment or protection, or an upward movement for scale or increase.	Use only when an identifiable schema can be named and no direct action, form, locus, route, or conventional symbol provides a better account. Abstractness alone is insufficient. If no defined mapping can be assigned reliably, use other.

4.2 High-priority boundary decisions

Potential overlap	Decision rule
Acting vs. Enacting	Specific object-directed manual manipulation was coded as acting. Broader bodily action, state, or experiential reenactment was coded as enacting.
Drawing vs. Path mapping	A contour or visible outline was drawing. A route, trajectory, or movement between locations was path mapping.
Embodying vs. Representing	If the handshape or configuration itself instantiated the referent form, the unit was embodying. If the hand mainly functioned as a token for an entity, it was representing.
Pointing vs. Locating	Indicating a locus was pointing. Placing a represented entity or component at a locus was locating.
Representing vs. Locating	Entityhood as the main contribution led to representing. Spatial placement as the main contribution led to locating.
Symbolic mapping vs. Enacting	A culturally recognizable form used for its conventional association was symbolic mapping. Reenactment of the corresponding activity was enacting.
Schema mapping vs. relation-based strategies	Concrete or imagined locus, placement, or trajectory was coded as pointing, locating, or path mapping. Schema mapping required a more abstract schematic relation such as balance, containment, opposition, scale, force, or cycle.
Schema mapping vs. Other	Schema mapping was assigned only when a specific schema could be identified. It was not a catch-all for abstract, unusual, or difficult gestures. Unresolved units were coded as other.

5. Classification of Combination Structure

Response-level combination structure was determined after segmentation. The criterion was the number and temporal organization of meaningful gesture units, not the number of strategy labels. Two or more semantically distinguishable units could therefore form a combination even when the units received the same iconic strategy label.

Response category	Operational rule	Illustrative coding
Non-combinatorial	One meaningful gesture unit. A bimanual production remained non-combinatorial when both hands jointly formed one integrated depiction.	Drawing
Sequential-only	Two or more meaningful units occurred in temporal succession and no simultaneous pairing of distinguishable units was present.	Drawing + Acting
Simultaneous-only	The two hands encoded distinguishable meaningful units within the same temporal interval and no larger sequential progression was present.	Acting & Embodying
Mixed	At least one simultaneous bimanual pairing occurred within a larger sequential progression.	Drawing & Embodying + Acting & Embodying

Working notation used “+” for sequential relations and “&” for simultaneous pairings. These symbols describe organization only and do not imply grammatical constituency.

6. Coding of Simultaneous and Sequential Organization

6.1 Focal and anchoring roles in simultaneous pairings

Each valid simultaneous pair was coded as containing a focal-hand unit and an anchoring-hand unit. These labels describe local functional positions within the pairing and do not refer to anatomical handedness, handedness preference, or stable specialization of either hand. The strategy category itself was not used to assign hand role.

Role	Operational cues
Focal-hand unit	Comparatively greater local movement prominence, a change produced against a more stable reference, or the unit organized relative to the paired unit.
Anchoring-hand unit	Comparatively greater temporal stability, maintenance of a local frame or reference, or the unit that provided the stable spatial relation against which the paired unit was organized.

Role assignment was made pair by pair from relative movement, temporal stability, and pairwise dependency. Consequently, distributions of strategies across focal and anchoring positions describe how those functionally coded positions were populated. They should not be interpreted as an independent test of anatomical hand specialization.

6.2 Derived variables for sequential organization

Variable	Rule
Sequential position	The first unit was initial, the last unit was final, and any intervening unit was medial. A two-unit sequence therefore had initial and final positions only.
Unordered co-occurrence pair	All strategy pairs occurring within the same sequential response were extracted without preserving order. For A + B + C, the pairs were A + B, A + C, and B + C.
Adjacent transition	Local temporal transitions preserved order. For A + B + C, the adjacent transitions were A to B and B to C.

6.3 Treatment of mixed responses

Mixed responses were counted once as mixed at the response level. For simultaneous analyses, every valid bimanual pairing was retained in full. For sequential analyses, each simultaneous interval was represented by its focal-hand unit so that the temporal progression contained one unit per step. The paired anchoring-hand unit remained represented in the simultaneous data and was not treated as an additional sequential unit.

For example, Drawing & Embodying + Acting & Embodying + Acting & Embodying contributed three simultaneous pairings. Its sequential progression was represented as Drawing + Acting + Acting.

7. Other-Coded Units and Exclusions

A unit was coded as other only when it appeared to contribute representational content but could not be assigned reliably to one of the 11 defined iconic strategies after the boundary rules above were applied. Other was therefore the residual category. Schema mapping was not used for this purpose.

- Use other when a unit is communicative but its representational strategy remains unclear, or when it is genuinely ambiguous between defined strategies with no dominant mapping that can be identified.
- Do not use other merely because a gesture is abstract, unusual, or difficult. If a defined action, form, relation, convention-based, or schema-based mapping is identifiable, assign that strategy.
- Other-coded units were excluded from structural analyses that required a valid strategy label, including strategy position, co-occurrence, transition, hand-role, and pair-structure analyses.
- Removing an other-coded unit from a structural dataset did not retroactively change the response-level screening status. If no valid analyzable component remained for a particular structural analysis, that response or pair did not enter that analysis.

8. Worked Examples and Scope of Interpretation

Response type	Target and gesture description	Coding decision
Non-combinatorial	Plate. The participant traces one circular outline in the air.	Drawing. One meaningful unit.
Sequential-only	Bread. The participant first traces an oval shape and then mimics cutting it.	Drawing + Acting. Two distinguishable units in succession.
Simultaneous-only	Mobile phone. One hand forms or holds an object-like shape while the other hand acts as if operating it.	Acting & Embodying. Two distinguishable units occur in the same temporal interval.
Mixed	Pizza. One hand maintains a flat base while the other first traces a circle, then performs cutting, then performs eating.	Drawing & Embodying + Acting & Embodying + Acting & Embodying. Simultaneous pairing is embedded in a sequential progression.

8.1 Scope of the coding labels

The coding scheme describes observable representational organization in manual silent gesture. It does not assume that the productions have the lexical, morphological, or grammatical status of elements in an established sign language. Sequential and simultaneous refer to the organization of meaningful manual units. Focal and anchoring refer to local functional roles within a simultaneous pair. Non-manual behavior was not coded as an independent meaningful unit in the analyses reported here.

Because symbolic mapping and schema mapping were introduced after recurrent productions were observed, results involving these categories should be read as data-informed exploratory descriptions. The explicit boundary rules in Sections 4 and 7 were used to prevent these categories, especially schema mapping, from absorbing units that were better classified under a pre-defined strategy or that remained unresolved.
